# Matrix-controlled iron bioavailability in unrefined whole-pulse low-moisture extrudates

**DOI:** 10.1016/j.crfs.2026.101589

**Published:** 2026-09-30

**Authors:** Mursalin Sajib, Jason Wiesinger, Hexiang Xie, Karen Cichy, Sharon Hooper, Kevin Howe, Raymond Glahn, Lutz Grossmann

**Affiliations:** a Department of Food Science, University of Massachusetts Amherst, Amherst, MA, USA; b Department of Life Sciences – Food and Nutrition Science, Chalmers University of Technology, Gothenburg, Sweden; c USDA-ARS, Robert W. Holley Center for Agriculture and Health, Ithaca, NY, USA; d USDA-ARS, Sugarbeet and Bean Research Unit, East Lansing, MI, USA; e Department of Food Science and Human Nutrition, Michigan State University, East Lansing, MI, USA

**Keywords:** Plant-based, Unrefined ingredients, Nutrition-sensitive crop selection, Bean flour, Low-moisture extrusion, Polyphenols, Flavonoids, Iron bioavailability, Iron deficiency anemia, Dietary iron, Ready-to-eat, Puffed snacks

## Abstract

Iron deficiency remains one of the most prevalent micronutrient deficiencies globally, disproportionately affecting populations that rely on plant-based diets. Although pulses are widely consumed staple foods and often contain substantial amounts of iron, iron concentration alone is a poor predictor of nutritional value because absorption is strongly constrained by the food matrix after processing these pulses. Here, we show that unrefined pulse composition exerts a dominant influence on cellular iron uptake in extruded foods, often outweighing the effects of total iron concentration and processing method. Using low-moisture extrusion to produce ready-to-eat foods from unrefined pulse flours, we observed wide variation in cellular iron uptake among pulse types despite comparable iron contents, with Caco-2 cell ferritin formation ranging from 0.81 ± 0.04 to 32.17 ± 0.31 ng ferritin/mg cell protein. This variation was most strongly associated with matrix composition: the highest iron uptake occurred when a low level of inhibitory flavonoids coincided with a low phytate-to-iron molar ratio and abundant enhancer flavonoids, whereas a low inhibitor level alone was not sufficient for high iron uptake. Extrusion improved cellular iron uptake only modestly relative to traditional porridge preparation. Together, these findings highlight matrix-controlled bioavailability as a key factor in plant-based iron delivery and identify yellow bean-based puffed snacks as a promising, consumer-friendly strategy for improving dietary iron intake.

## Highlights

• Unrefined pulse flours were extruded into shelf-stable, ready-to-eat puffed snack.
• In whole-pulse extrudates, matrix composition governed cellular iron uptake.
• Low inhibitory-flavonoid content, not iron concentration, tracked high iron uptake.
• Lower phytate correlated with more iron uptake among the different pulse types.
• Yellow bean puffs showed the highest cellular iron uptake among the pulses tested.

## Graphical abstract

**Figure:**
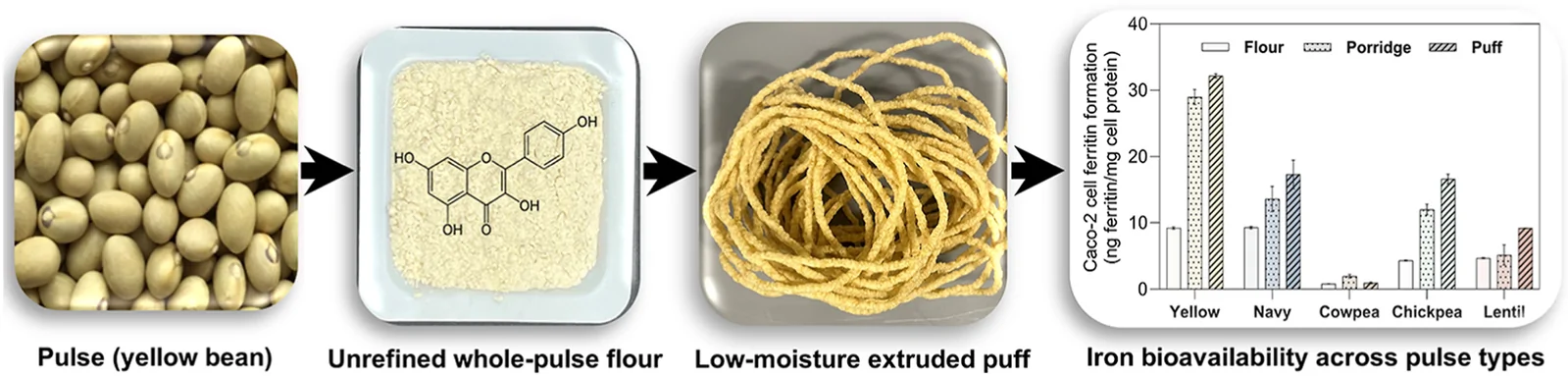

## 1 Introduction

Iron deficiency remains one of the most prevalent micronutrient deficiencies worldwide and is a leading cause of anemia, contributing substantially to global morbidity and years lived with disability (Gardner et al., 2023; Lopez et al., 2016; Pasricha et al., 2021; Vos et al., 2017; Zimmermann and Hurrell, 2007). Iron is an essential mineral required for hemoglobin synthesis and oxygen transport in red blood cells (NIH, 2023); however, the nutritional value of iron in foods is determined not only by its concentration but, critically, by its bioavailability. Insufficient absorption of dietary iron disrupts erythropoiesis, leading to iron deficiency and, in severe cases, iron deficiency anemia (Miller, 2013). Children and women are particularly vulnerable due to elevated physiological iron requirements, estimated at 7–10 mg per day for children, 18 mg per day for non-pregnant women, and 27 mg per day during pregnancy (Lopez et al., 2016; NIH, 2023). Although iron deficiency anemia affects populations in both low- and high-income regions, prevalence remains highest among women and children, especially in developing countries (DeMaeyer and Adiels-Tegman, 1985; WHO, 2001; Zimmermann and Hurrell, 2007). More recent evidence suggests iron deficiency affects a large proportion of American adults as well (Barton et al., 2024; Tawfik et al., 2024).

Low iron bioavailability represents a dominant constraint on effective iron delivery in populations that rely heavily on plant-based diets, where absorption of non-heme iron often remains below 10% (Miller, 2013; Zimmermann et al., 2005; Zimmermann and Hurrell, 2007). This challenge has gained increasing relevance amid global dietary shifts toward plant-based protein sources, driven in part by environmental sustainability considerations (Sadohara et al., 2026; van Wonderen et al., 2023). Importantly, foods may contain substantial quantities of iron without providing any iron that is readily absorbed by the human upper intestine. Iron bioavailability is governed by both the chemical form of iron and the surrounding food matrix, which modulates iron solubility, redox state, and interactions with dietary components (Piskin et al., 2022). Whereas heme iron from animal-derived foods is believed to be efficiently absorbed and relatively insensitive to dietary composition, non-heme iron from plant sources, especially from beans and other pulse crops, exhibits lower and more variable bioavailability and is highly responsive to matrix effects (Glahn et al., 2025; Wiesinger et al., 2022).

Non-heme iron occurs predominantly in the oxidized ferric (Fe^3+^) form and must be reduced to ferrous (Fe^2+^) iron prior to uptake by intestinal enterocytes, rendering absorption sensitive to dietary enhancers and inhibitors (Piskin et al., 2022). Certain polyphenols, such as kaempferol, have been shown to enhance iron uptake in Caco-2 cell models (Hart et al., 2015, 2017; Li et al., 2025), whereas inhibitors such as phytic acid and some polyphenols form stable complexes with ferric iron that limit absorption (Hart et al., 2015; Piskin et al., 2022). These interactions highlight that iron bioavailability in plant-based foods is governed not by iron content alone but by matrix-level and processing constraints that can either promote or restrict intestinal iron uptake (Glahn et al., 2025; Sadohara et al., 2025; Wiesinger et al., 2022).

A range of technological strategies has been developed to enhance iron bioavailability, including encapsulation with starch (Bryszewska et al., 2019) or liposomes (de Alvarenga Antunes et al., 2020), chelation with dairy proteins (Sabatier et al., 2020; Shilpashree et al., 2020), and, more recently, engineered oat protein nanofibril-iron hybrid systems (Zhou et al., 2025). Although these approaches have demonstrated improved iron bioavailability in animal models or human trials, they may be insufficient to mitigate iron deficiency at scale. In particular, large-scale iron fortification in developing countries is constrained by storage stability, processing complexity, and economic feasibility, while sensory acceptability remains a challenge across both developing and developed regions (Hurrell et al., 2004; Hurrell, 2002, 2022; Osendarp et al., 2018). These limitations underscore the need for scalable strategies capable of improving iron status in vulnerable populations.

In this context, ready-to-eat foods produced from unrefined whole-pulse flours using low-moisture extrusion offer a promising pathway for plant-based iron delivery. The use of unrefined whole-pulse flours enables retention of intrinsic micronutrients and polyphenols, such as seed-coat polyphenols that may enhance iron bioavailability in the context of this present study (Hart et al., 2019), as well as improve colon function (Bruce et al., 2000), while avoiding fractionation steps that generate side streams, thereby contributing to more sustainable food production systems (Germerdonk et al., 2025). Moreover, low-moisture extrusion provides a robust processing platform capable of accommodating the inherent chemical and physical complexity of whole plant ingredients and converting unrefined pulse flours into shelf-stable, ready-to-eat foods (Germerdonk et al., 2025). However, as with many plant-based foods, iron bioavailability in pulse-based products remains strongly constrained by the food matrix. Addressing this challenge requires an integrated understanding of pulse composition, including intrinsic iron concentrations, the levels of iron-inhibiting compounds such as phytic acid, and the presence of polyphenols that may either enhance or inhibit iron absorption (Hooper et al., 2023; Wiesinger et al., 2020). In addition, processing conditions such as low-moisture extrusion may further modulate iron bioavailability by altering matrix structure, reducing inhibitors, and modifying polyphenol profiles, and therefore warrant systematic investigation (Akande et al., 2017; Anton et al., 2009; Glahn et al., 2025; Sánchez-Quezada et al., 2024; Wiesinger et al., 2020, 2023; Yadav et al., 2019).

Accordingly, this study investigates the extrudability of unrefined whole-pulse flours from three specialty pulse cultivars grown in the United States, selected for their relevance to promoting the use of locally produced beans in food product development. Two commercially available pulse flours (chickpea and lentil) were also included for comparison. Using low-moisture extrusion, these flours were processed into ready-to-eat puffed snacks, while simultaneously examining the respective roles of pulse type and processing type in influencing potential iron bioavailability. By relating mineral, phytate, and flavonoid composition to potential iron bioavailability using correlation and principal component analyses, this work identifies key constraints and opportunities for improving plant-based iron delivery through the use of unrefined pulse-based ingredients, with potential relevance for mitigating iron deficiency at scale.

## 2 Materials and methods

### 2.1 Materials

Three dry bean cultivars used in this study were *Phaseolus vulgaris* L. USDA Yellowjacket, a Manteca yellow bean, grown in Michigan in 2024 (referred to as “Yellow” throughout the manuscript); a commercial navy bean cultivar mix, grown in Michigan in 2024 (“Navy”); and *Vigna unguiculata* (L.) Walp. California Blackeye 77 cowpea (Huynh et al., 2022), grown in California in 2024 (“Cowpea”). Whole seeds of each cultivar were roasted in a convection oven at 110 °C for 70 min and subsequently milled using a compression–decompression (C/D) milling system (Enagon, LLC, Saugatuck, MI, USA). The C/D milling process generated fine particle whole-pulse flours with variable protein and fiber concentrations. Chickpea (whole chickpea flour; One in a Mill, Elizabeth, NJ, USA) and red lentil (red lentil flour; Food to Live, Brooklyn, NY, USA) flours were purchased from an online marketplace and used as reference materials. All flours were stored at −20 °C until further use. The proximate composition of all flours is reported in Table 1.

**Table 1:** Proximate composition and fiber concentrations of different pulse flours used to produce porridges and expanded puff products.

		Yellow	Navy	Cowpea	Chickpea	Red lentil
Moisture (%)		4.08 ± 0.06^c^	2.52 ± 0.02^d^	1.41 ± 0.04^e^	6.89 ± 0.0^b^	8.21 ± 0.08^a^
Protein (%)		16.98 ± 0.09^e^	17.72 ± 0.07^d^	21.85 ± 0.04^b^	20.73 ± 0.05^c^	26.54 ± 0.18^a^
Total starch (%)		44.56 ± 0.76^a^	32.81 ± 2.34^a^	39.51 ± 4.33^a^	40.36 ± 2.54^a^	43.67 ± 7.80^a^
Crude lipid (%)		0.72 ± 0.62^b^	0.81 ± 0.76^b^	0.97 ± 0.19^b^	5.96 ± 0.05^a^	0.55 ± 0.39^b^
Dietary fiber (%)	Soluble	3.63 ± 0.35^a^	0.87 ± 0.61^b^	4.03 ± 0.16^a^	3.60 ± 0.64^a^	0.70 ± 0.28^b^
	Insoluble	16.14 ± 0.85^a^	18.43 ± 0.67^a^	9.33 ± 0.30^b^	7.67 ± 0.16^bc^	6.89 ± 0.71^c^
	Total	19.77 ± 0.49^a^	19.30 ± 0.06^a^	13.36 ± 0.14^b^	11.27 ± 0.48^c^	7.59 ± 0.43^d^

Different superscript letters within the same row indicate significant differences (*p* < 0.05). Results are reported on an as-is basis. For protein analysis, a nitrogen-to-protein conversion factor of 6.25 was used.

### 2.2 Proximate composition analysis

Moisture content was measured in accordance with the NREL Laboratory Analytical Procedure (NREL/TP-510-42621) by drying flour samples in a vacuum oven at 105 °C until constant weight (Sluiter et al., 2008). Protein content was determined using the Dumas combustion method with a rapid N exceed analyzer (Elementar, Langenselbold, Germany), applying a nitrogen-to-protein conversion factor of 6.25 (Mariotti et al., 2008). Total starch content was determined using a commercial starch assay kit (K-TSTA-100A; Megazyme, Bray, Ireland) according to the RTS-NaOH procedure, following the manufacturer's protocol. Crude lipid content was determined gravimetrically after evaporation of an aliquot of the chloroform phase from a chloroform-methanol (1:1, v/v) extraction (Lee et al., 1996). Total dietary fiber measurements (soluble and insoluble) were conducted commercially at Great Lakes Scientific, Inc. located in Stevensville, Michigan (www.glslab.com).

### 2.3 Production of extruded puffs

Each flour was extruded individually using a co-rotating twin-screw extruder equipped with a volumetric powder feeder (Three-Tec GmbH, Seon, Switzerland) operated at a throughput of 0.8 kg/h, a screw speed of 300 rpm, and fitted with a 1 mm die hole. The extruder had a length-to-diameter (L/D) ratio of 40:1 and was configured with 19 screw elements arranged sequentially as forward conveying elements, followed by 60° and 90° kneading elements, and concluding with forward conveying elements (Fig. 1). Extrusion processing was performed using a barrel temperature profile of 8–50–80–100–120–140–160 °C. The extrusion moisture content was set at 20%, with water addition adjusted based on the initial flour moisture. Two independent extrusion trials were conducted for each flour type, and the resulting puffs were vacuum-sealed and stored at either 4 °C or −20 °C until further analysis.

**Fig. 1:**
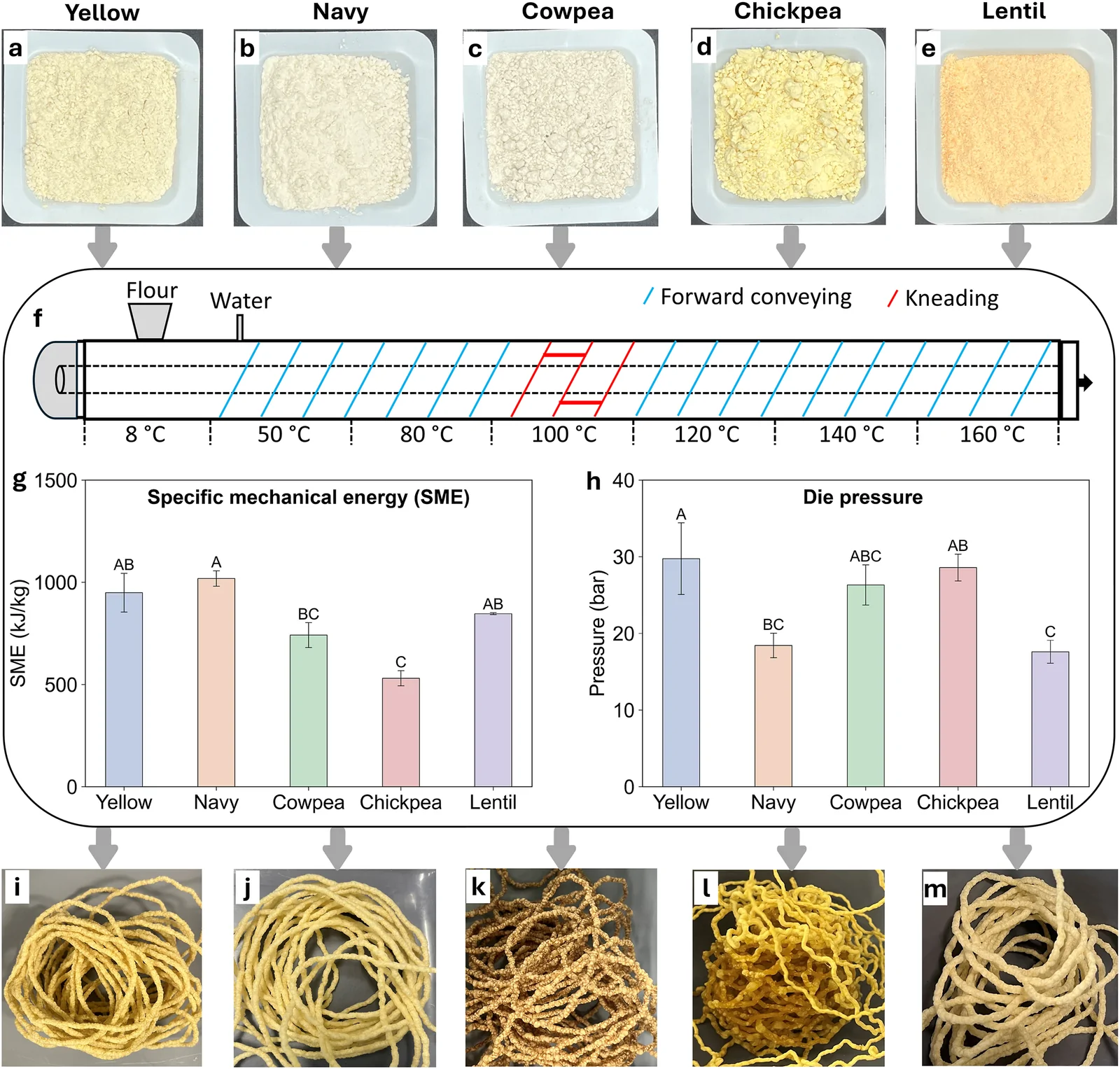
Visual representations of pulse flours (a–e), extrusion screw configuration and barrel temperature profile (f), specific mechanical energy (SME) and die pressure recorded during low-moisture extrusion of different pulse flours (g, h), and representative images of the resulting extruded puffs (i–m). Results are reported on an as-is basis. Significant differences (*p* < 0.05) are denoted by different uppercase letters.

### 2.4 Extrusion process characteristics

To assess the extrudability of each flour under the tested extrusion conditions (described above), specific mechanical energy (SME) and die pressure were compared. SME was calculated according to Stehle et al. (2024) using equation (1), where *n* denotes screw rotation speed (rpm), *M* denotes screw torque (Nm), and *m* represents total throughput (kg/min). Values for *n*, *M*, and die pressure were obtained from the extruder log data.

(equation 1) $$\text{SME}=(\frac{2\pi nM}{m})/1000\;\left(\text{kJ}/\text{kg}\right)$$

### 2.5 Physical and textural properties of extruded puffs

***Expansion ratio****:* The method of Maskus and Arntfield (2015) was used to calculate the expansion ratio of the extruded puffs using equation (2), based on a die hole diameter of 1 mm. Ten randomly selected puffs from each extrusion trial were measured, and the mean value was treated as a single replicate.

(equation 2) $$\text{Expansion}\;\text{ratio}=\frac{\text{Puff}\;\text{diameter}}{\text{Extruder}\;\text{die}\;\text{hole}\;\text{diameter}}$$

***Hardness****:* The hardness of the puffs was measured according to Luo et al. (2020) using a TA.XT.plus texture analyzer (Stable Micro Systems, Surrey, UK) equipped with a 5 kg load cell and a 1 mm Warner-Bratzler shear blade. Tests were conducted on puffs cut to 25 mm in length at a test speed of 0.10 mm/s, with samples positioned perpendicular (90°) to the blade. Four randomly selected puffs from each extrusion trial were analyzed, and the maximum peak force recorded for each puff was defined as hardness. The mean of the four measurements was considered as a single replicate.

***Water activity****:* The *a*_w_ of the puffs, used as an indicator of crispiness, was measured using an AquaLab 4 TE Dew Point Water Activity Meter (Pullman, WA, USA) after calibration with external water activity standards (METER Group, Pullman, WA, USA).

### 2.6 Preparation of porridge

A 150 mL beaker containing 50 mL of distilled water was first brought to a boil on a Corning® PC-420 Hot Plate Stirrer set to high heat (setting 10). While the water was heating, 15 mL of distilled water was added to a separate 150 mL beaker containing 5 g of whole-pulse flour. The mixture was gradually dissolved under constant stirring to form a slurry and stirred at room temperature for a total of 3 min at a moderate speed (setting 5–6) to ensure uniform consistency. The slurry was carefully added to the boiling water, and the mixture was allowed to boil at high heat (setting 9-10) for 4 min with continuous stirring (setting 7-8). After boiling, the heat was set to the lowest setting 1, and the porridge was simmered uncovered for an additional 6 min with constant stirring. Following cooking, the porridge was carefully transferred into polypropylene 50 mL centrifuge tubes and allowed to cool at room temperature with the caps removed for 10–15 min. The volume of each porridge sample was estimated using the volume markers on the side of each tube. The tubes were then tightly capped and weighed to determine total wet weight. The tubes were positioned sideways (horizontally) to increase the surface area of the porridge before being stored at −80 °C. Porridge samples were freeze-dried (VirTis® Research Equipment, Gardiner, NY, USA) and then tightly capped before weighing to determine total dry weight. The dried porridge was manually crushed into a homogeneous powder using a clean glass rod. The powdered porridge samples were stored at −20 °C until further analysis.

### 2.7 Phytate analysis

The total phytate (phytic acid) concentrations in flours, porridges, and extruded puffs were quantified using a commercial phytate/total phosphorus assay kit (K-PHYT; Megazyme® International, Bray, Ireland) following an extraction procedure adapted from Wiesinger et al. (2020). Samples (500 mg; dry weight) were extracted with 0.66 M hydrochloric acid (10 mL) at ambient temperature for 16 h, centrifuged at 16,000 × g for 10 min, and the supernatant was neutralized with 0.75 M sodium hydroxide prior to storage at −20 °C. Phytate-derived phosphorus released by enzymatic digestion was quantified spectrophotometrically at 655 nm, and phytic acid concentration was calculated using MegaCalc™ after correction for free inorganic phosphate.

### 2.8 Lectin analysis

Hemagglutination activity was assessed using a modified procedure based on Campion et al. (2009) and Nciri et al. (2015). Proteins were extracted from 100 mg (dry weight) of sample in 1.5 mL of phosphate-buffered saline (PBS; pH 7.4) by gentle shaking at 4 °C for 16 h, followed by centrifugation at 1500 x g for 5 min. The supernatant was serially diluted twofold in a 96-well microtiter plate over 11 successive dilution steps. Each dilution was mixed with 100 μL of a 1% (v/v) suspension of rabbit red blood cells (88R-R001; Fitzgerald Industries International) and incubated for 2 h at room temperature. Hemagglutination activity was evaluated visually. Compact erythrocyte sedimentation was recorded as no detectable lectin activity (nd), whereas positive hemagglutination was expressed as the reciprocal of the highest dilution showing visible agglutination, with higher titers indicating greater lectin activity (Campion et al., 2009; Nciri et al., 2015). Although thresholds for ‘high’ and ‘low’ lectin activity are not standardized, positive agglutination at high dilutions (e.g., ≥1:256) is generally associated with substantial lectin activity, while very low titers (≤1:2) or absence of agglutination indicate negligible activity following processing (Adamcová et al., 2021; Nciri et al., 2015; Thompson et al., 2025).

### 2.9 Mineral analysis

Mineral content in flours, porridges, and extruded puffs was determined following the method described by Hooper et al. (2023). The following elements were quantified: iron (Fe), zinc (Zn), copper (Cu), manganese (Mn), molybdenum (Mo), calcium (Ca), magnesium (Mg), potassium (K), phosphorus (P), sulfur (S), and aluminum (Al). Samples were oven-dried to constant weight (100 °C, 48 h), and 500 mg of the dried material was pre-digested in a concentrated acid mixture and subsequently diluted to 20 mL with ultrapure water before analysis by inductively coupled plasma-atomic emission spectroscopy (ICP-AES; Thermo iCAP 6500 Series, Thermo Scientific, Cambridge, UK). For digestion, samples were treated with 3 mL of a nitric acid-perchloric acid mixture (60:40, v/v) and held at ambient temperature for 16 h, followed by stepwise heating in a digestion block to 120 °C and then 145 °C, with an intermediate addition of 2 mL concentrated nitric acid, before final heating to 190 °C until the samples evaporated to dryness. Digests were cooled to room temperature prior to dilution with water. Yttrium (0.5 μg/mL; 10M67–1) was used as an internal standard, and quality control standards (High Purity Standards, SC, USA) were analyzed every 10 samples. Digestion methods and ICP-AES measurements were verified using Standard Reference Material® (Typical Diet 1548b; National Institute of Standards and Technology NIST, Gaithersburg, MD, USA).

### 2.10 Flavonoid analysis

#### 2.10.1 Flavonoid solvents and standards

For solvents, Optima™ LC/MS grade acetone, formic acid, methanol, and water, as well as HPLC grade dimethyl sulfoxide were purchased from Fisher Scientific (Fair Lawn, NJ, USA). Chromasolv™ LC-MS grade hexane was procured from Honeywell Riedel-de Haën™ (Seelze, Germany). For standards, Cinnamtannin B1 analytical standard was purchased from Cayman Chemical (Ann Arbor, MI, USA). Primary grade standards for cyanidin-3-O-glucoside chloride (chrysanthemin), (−)-epicatechin, (−)-epigallocatechin, (−)-gallocatechin, kaempferol, myricetin, procyanidin A2, procyanidin B1, procyanidin B2, procyanidin C1, quercetin, and quercetin-3-O-glucoside (isoquercitrin) were purchased from Chromadex® (Longmont, CO, USA). Analytical grade standards for DL-catechin, malvidin-3-O-glucoside chloride (oenin), pelargonidin-3-O-glucoside chloride (callistephin), rutin, and reagent grade delphinidin-3-O-glucoside chloride (myrtillin) were also purchased from Chromadex®. Petunidin-3-O-glucoside chloride was purchased from Extrasynthese (Genay, France). Kaempferol-3-O-glucoside (astragalin), kaempferol 3-sambubioside(leucoside), and myricetin-3-O-glucoside were purchased as analytical standards from INDOFINE Chemical Company, Inc. (Hillsborough, NJ, USA). Analytical grade standards for (+)-afzelechin and (−)-epiafzelechin are from AOBIOUS, Inc. (Gloucester, MA, USA). Cinnamtannin A2 was obtained from Phytolab (Vestenbergsgreuth, Germany) as phyproof® analytical standard.

#### 2.10.2 Flavonoid extraction

For extraction, duplicate ∼300 mg samples of freeze-dried flour, porridge, and expanded puffs were added to pre-weighed 2.0 mL microcentrifuge tubes. Initial extraction begins with the addition of 1 mL methanol:water:formic acid (80:12:2) followed by 30 min of sonication in a 4 ^°^C cold room. Samples are then placed on a rocker for 3.5 h at 4 ^°^C. The initial extraction was centrifuged at 16,000 x g for 6 min to pellet debris and the supernatant was transferred to a separate 2.0 mL microcentrifuge tube. Supernatants were dried using a Speed Vacuum Concentrator (Labconco, Kansas City, MO, USA) modified with a cold finger trap containing dry ice and ethanol for condensation of organic solvent vapors. The second extraction involves the resuspension of the pellet in 1 mL of methanol:formic acid (98:2). Samples were again placed on a rocker for an additional 3 h at 4 ^°^C. The second extraction was centrifuged at 16,000 x g for 6 min to pellet debris, while the supernatant was removed and pooled into the dried extract tube containing the first extraction of each sample. The pooled extracts were again dried in a Speed Vacuum Concentrator. A third extraction was performed with the addition of 1 mL acetone:water:formic acid (70:28:2) to the resuspended pellet. Samples were then placed on a rocker in the cold room (4 ^°^C) overnight. Debris was pelleted at 16,000 x g for 6 min, and the supernatants were transferred for a third time to the pooled extract tube and dried in a Speed Vacuum Concentrator. To remove any oily or viscous residues that were observed in some of the extracts, 0.75 mL of methanol:water (50:50) was added to each tube along with 0.25 mL of hexane. Samples were agitated on a rocker in the cold room for 1.5 h, then centrifuged at 16,000 x g for 6 min. The hexane phase was removed from each sample with a fine glass pipette and discarded. The remaining methanol:water phase was evaporated to dryness in the Speed Vacuum Concentrator. Dried extracts were stored at −20 °C. For analysis, samples were removed from freezer and reconstituted with 0.25 mL dimethyl sulfoxide. Samples were then sonicated at room temperature for 15 min and given a final centrifugation at 16,000 g for 6 min before transferring to an autosampler vial.

#### 2.10.3 Flavonoid analysis (UPLC-MS)

Quantification of individual flavonoids was performed with LC-MS on a Waters™ H-Class Plus UPLC system coupled with a QDa single-quadrupole MS detector (Waters™, Manchester, UK). Individual flavonoid standards were initially evaluated via infusion of commercial standards into mobile phase flow and assigned a *m/z* value in either positive or negative ionization mode based on selectivity and sensitivity of observed ions. Injection tests were then performed to determine retention times for those *m/*z values, which were compiled into a scheduled selected ion recording method. Dilutions of commercial standards were used to establish calibration ranges and fits for the individual flavonoid analytes. The method parameters are described in Table 2. An Acquity BEH C18 2.1 × 100 mm column with 1.7 μm particles (Waters™) held at 60 °C was used for the separation at a flow rate of 500 μL/min. Mobile phase A was water and mobile phase B was methanol, both containing 0.1% formic acid. The mobile phase gradient was 0 → 3.5 min, 5 → 30% B; 3.5 → 9 min, 30 → 60% B; 9 → 9.5 min, 60 → 90% B; 9.5 → 10 min, 90 → 5% B; followed by 5% B hold to 13.5 min. For all acquisitions a probe temperature of 600 °C, capillary voltage of 800 V, cone voltage of 15 V, and sampling rate of 5 Hz were used. Individual 4 mM stock solutions of each flavonoid standard were prepared in DMSO and used to prepare dilutions of standards. Although all positive and negative ions are collected in a single injection for extracted samples, separate calibration curves for positive and negative ions were prepared to facilitate dilutions for higher concentration points. Individual calibration points at 1, 2.5, 5, 10, 25, 50, 100, and 150 pmol (injected) were collected for all analytes even if they are not used in the calibration fit for each analyte. To monitor quality, calibration standards were injected before and after the extracted samples using the same analytical method. Injection volume was 2 μL. MassLynx v4.2 software was used for analysis and TargetLynx XS v4.2 was used for quantitation (Waters™).

**Table 2:** Flavonoid acquisition parameters.

Analyte	Ionization mode	*m/z* Monitored	Retention time (min)	Calibration range (pmol)	Calibration fit
(−)-Gallocatechin	negative	305.0	1.17	1-100	linear
Procyanidin B1	negative	577.2	1.96	2.5-150	linear
(−)-Epigallocatechin	negative	305.0	2.04	5-150	linear
DL-Catechin	negative	289.1	2.15	2.5-150	linear
Delphinidin-3-O-glucoside	positive	465.1	2.38	2.5-100	linear
Procyanidin B2	negative	577.2	2.55	10-150	linear
Cinnamtannin B1	negative	863.3	2.74	5-150	linear
(+)-Afzelechin	negative	273.0	2.78	5-150	linear
Cyanidin-3-O-glucoside	positive	449.2	2.87	2.5-150	linear
(−)-Epicatechin	negative	289.1	3.00	5-150	linear
Procyanidin C1	positive	867.3	3.02	2.5-150	linear
Petunidin-3-O-glucoside	positive	479.1	3.05	2.5-150	linear
Cinnamtannin A2	negative	576.6	3.06	5-100	linear
Pelargonidin-3-O-glucoside	positive	433.1	3.28	2.5-100	linear
(−)-Epiafzelechin	negative	273.0	3.61	2.5-100	linear
Malvidin-3-O-glucoside	positive	493.2	3.62	2.5-100	linear
Procyanidin A2	positive	577.2	3.93	2.5-150	quadratic
Myricetin-3-O-glucoside	positive	503.1	3.94	2.5-100	quadratic
Quercetin-3-O-rutinoside	negative	609.3	4.66	1-100	quadratic
Quercetin-3-O-glucoside	positive	487.1	4.66	1-100	quadratic
Kaempferol 3-sambubioside	negative	579.2	4.68	2.5-100	quadratic
Myricetin	positive	319.0	5.00	2.5-100	linear
Kaempferol-3-O-glucoside	positive	471.2	5.30	5-150	quadratic
Quercetin	positive	303.0	6.15	1-150	linear
Kaempferol	positive	287.0	7.11	5-150	linear

### 2.11 Cellular iron uptake (Caco-2 cell bioassay)

An established *in vitro* digestion/Caco-2 cell culture model of the human intestinal epithelial barrier was used to assess the potential iron bioavailability of each flour, porridge and puff sample. The bioassay was performed according to the detailed methods described in Glahn et al. (1998) and Glahn (2022).

#### 2.11.1 Caco-2 cell culture

Caco-2 cells (HTB-37, American Type Culture Collection®, Gaithersburg, MD, USA; obtained at passage 21) were seeded at passage 35–38 at a density of 50,000 cells/cm^2^ in 6-well collagen-coated plates (3506, Costar®, Cambridge, MA, USA). Cells were cultured for 13 days at 37 °C in an incubator with a 5% CO_2_ air atmosphere (constant humidity) using Dulbecco's modified Eagle's medium (DMEM; 12100046, Gibco, Grand Island, NY), which was supplemented with 25 mM HEPES (pH 7.2; H3375, Sigma Aldrich Co., St. Louis, MO, USA), 10% (v/v) fetal bovine serum (Optima; S12450, GeminiBio, West Sacramento, CA, USA), and 1% (v/v) antibiotic-antimycotic solution (ZellShield® 13-0050, Minerva Biolabs, Germany). The DMEM medium in the 6-well plates and large cell culture flasks used for seeding was changed every 48 h. Twenty-four hours prior to each bioassay, the culture medium was replaced with iron-free Minimum Essential Medium (MEM [pH 7]; 41500034, Gibco) supplemented with 10 mM PIPES (piperazine-N,N′-bis-[2-ethanesulfonic acid; P3768, Sigma Aldrich Co.), 1% antibiotic-antimycotic solution, hydrocortisone (4 mg/L; H0396, Sigma Aldrich Co.), insulin (5 mg/L; I2643, Sigma Aldrich Co.), selenium (5 μg/L; S5261, Sigma Aldrich Co.), triiodothyronine (34 μg/L; T6397, Sigma Aldrich Co.), and epidermal growth factor (20 μg/L; E4127, Sigma Aldrich Co.). Cells were always covered with a fresh 1 mL aliquot of MEM (pH 7) during each experiment.

#### 2.11.2 Preparation of pepsin and pancreatin-bile solutions

On the day of the experiment, a pepsin solution was prepared by dissolving 0.145 g of pepsin (P6887, Sigma Aldrich Co.) in 50 mL of 0.1 M HCl and gently mixed on a platform shaker for 30 min at room temperature. For the pancreatin-bile solution, 0.35 g of pancreatin (P1750, Sigma Aldrich Co.) and 2.1 g of bile extract (glycine and taurine conjugates of hyodeoxycholic and other bile salts; B8631, Sigma Aldrich Co.) were solubilized in 175 mL of 0.1 M NaHCO_3_. To remove contaminants, including iron from the pancreatin-bile extracts, 87.5 g of Chelex 100 (cation exchange resin; 1422832, Bio-Rad Laboratories Inc., Hercules, CA, USA) was added to the pancreatin-bile solution and mixed by shaking for 30 min at room temperature. The slurry was poured into a 1.5 cm diameter chromatography column (Kimble® Flex-Column® 420400-1530, DWK Life Sciences, Vineland, NJ, USA) to remove the Chelex and eluted with an additional 70 mL of 0.1 M NaHCO_3_, collecting this volume into the pancreatin-bile solution.

#### 2.11.3 In vitro digestion

Porridge and puff samples were prepared as described above and freeze-dried prior to digestion; cellular iron uptake is therefore expressed on a freeze-dried weight basis. A 0.5 g milled sample of freeze-dried flour, porridge or puff was first weighed in triplicate into a 50 mL polypropylene centrifuge tube (Snap-Pop lid; 229498, CELLTREAT® Scientific Products, Ayer, MA, USA), followed by the addition of 10 ml of physiological saline, pH 2 (140 mM NaCl and 5 mM KCl). Samples were mixed and re-adjusted to pH 2 by the addition of small amounts of 1 M HCl. Gastric digestion was initiated by adding 0.5 mL of prepared pepsin solution to each sample and incubating on a rocking shaker at a low, gentle setting for 1 h at 37 °C. During gastric digestion, a sterilized insert ring fitted with a dialysis membrane (SpectraPor 7, 15,000 MWCO; 132123, Spectrum Laboratories Inc., Milpitas, CA, USA) was placed into each well of the 6-well plate with monolayers of Caco-2 cells, creating a two-layer chamber system (insert rings were prepared the day before and stored in 18 MΩ water at 4 °C). Plates with inserts were returned to the CO_2_ incubator after a fresh 1 mL of iron-free MEM (pH 7) was added to each well. A simulated intestinal digestion was initiated by first adjusting the pH of each sample to 5.5 – 6.0 with 1 M NaHCO_3_. A 2.5 mL aliquot of the pancreatin-bile solution was added to each sample and the pH further adjusted to 6.9 – 7.0 with 1 M NaHCO_3_. Once the pH was adjusted, the volume of each tube was equalized with physiological saline (140 mM NaCl, 5 mM KCl, pH 6.7) by measuring the weight of the tube with a target value of 15 g. After carefully mixing each tube, a 1.5 mL aliquot of the intestinal digest was gently added to the upper chamber of each well to initiate the bioassay. The plate was covered and incubated for 2 h at 37 °C (5% CO_2_ air atmosphere) on a rocking shaker at 6 oscillations/min. The digest and insert ring were removed after the bioassay was terminated. An additional 1 mL of iron-free MEM (pH 7) was added to each well of Caco-2 monolayers. The cell culture plates were further incubated for 22 h in the incubator (37 °C; 5% CO_2_ air atmosphere), after which the cells were carefully rinsed twice with 2 mL of buffered saline (130 mM NaCl, 5 mM KCl, 5 mM PIPES, pH 6.7) before being lysed with sterilized water and harvested for analysis.

#### 2.11.4 Caco-2 cell ferritin formation (iron bioavailability)

The bioassay works according to the following principle: in response to increases in cellular iron concentrations after exposure to an *in vitro* digestion of a milled food sample, Caco-2 cells produce more ferritin protein; therefore, cellular iron uptake was determined as the increase in Caco-2 cell ferritin production expressed as a ratio to total Caco-2 cell protein (ng ferritin per mg of total cell protein) after exposure to a digested food sample for 2 h. Ferritin was measured the following day by enzyme-linked immunoassay (Human Ferritin ELISA kit, FRR31-K01, Eagle Biosciences Inc., Nashua, NH, USA) and total cell protein concentrations were quantified using the Bio-Rad DC™ protein assay kit (Bio-Rad Laboratories Inc., Hercules, CA, USA).

To confirm the responsiveness of the bioassay, and to ensure quality control between several weeks of the bioassay, each experiment was run with several food reference standards. These include a blank digest, which is only the physiologically balanced saline and the gastrointestinal enzymes. The blank digest was used to ensure there was no iron contamination in the bioassay. Ferritin values over the course of three cell culture experiments in Caco-2 cells that were exposed to the blank-digest are shown in Table 3. A cooked, lyophilized and milled red (cv. Red Hawk) and white (cv. Snowdon) kidney bean control was run with each assay to monitor the influence of high flavonoid content (red kidney bean) and no flavonoid content (white kidney bean) on the iron bioavailability of cooked whole beans (Table 3). In addition, a cooked, lyophilized and milled white kidney bean pasta (100% bean flour; cv. Snowdon) was run with each bioassay as a way to examine the effects on iron bioavailability in an extruded flour matrix of broken cotyledon cell walls, similar to the flour matrix in the pulse flours used in this study (Glahn et al., 2025). A sample of cooked, freeze-dried and milled ground beef (80% lean, 20% fat), as well as a sample of cooked, freeze-dried and milled Impossible™ plant-based burger were included in the Caco-2 bioassay as references for complex ingredient foods that are high in bioavailable iron and readily available to consumer (Table 3)

**Table 3:** Ferritin values for the different types of reference standards used to monitor the responsiveness of the Caco-2 cells over the course of three separate experiments.

Reference standards for the *in vitro* digestion/Caco-2 bioassay	Caco-2 Ferritin formation (ng/mg total cell protein)^a^
Blank Digest (no food)	2.47 ± 0.35^e^
Red kidney bean (cooked)	3.76 ± 0.44^d^
White kidney bean (cooked)	8.36 ± 0.51^c^
White kidney bean pasta (boiled)	24.05 ± 1.32^b^
Ground beef (cooked)	43.95 ± 2.73^a^
Impossible™ meat (cooked)	48.55 ± 3.31^a^

Different superscript letters within the same column indicate significant differences (*p* < 0.05).

a Ferritin protein measured in Caco-2 cells after exposure to an *in vitro* digestion of cooked and freeze-dried food products.

### 2.12 Statistical analysis

Results are presented as mean ± standard deviation (SD) from two independent extrusion trials per flour type (n = 2). Sample from each trial was analyzed in duplicate, and the average of these analytical replicates was treated as a single value; all statistical analyses were therefore based on n = 2 independent (processing) replicates per treatment. The study followed a 5 × 3 factorial design, with pulse type (Yellow, Navy, Cowpea, Chickpea, Lentil) and processing type (flour, porridge, puff) as factors. Data were evaluated by one-way or two-way ANOVA as appropriate, with Tukey's HSD post hoc test (α = 0.05). One-way ANOVA was used for single-factor variables (Fig. 1, Fig. 2), and two-way ANOVA for the effects of pulse type, processing type, and their interaction (Fig. 3, Fig. 4, Fig. 5). Full ANOVA summaries and effect sizes (η^2^, partial η^2^, ω^2^) are provided in Table S3–S12. Treatment means are shown with lowercase letters denoting differences among processing types within a pulse and uppercase letters denoting differences among pulses within a processing type. These groupings are presented for descriptive comparison and should be interpreted alongside the full factorial results, given that the interaction was significant in several cases.

Relationships between compositional variables and cellular iron uptake were examined by Spearman rank correlation (reported as Spearman's rank correlation coefficient, ρ) and principal component analysis. Spearman correlation was used given the small sample size and the non-normal, zero-inflated distribution of several flavonoid variables, and correlations were considered significant at *p* < 0.05. Both analyses were run on a full dataset (all individual minerals, phytic acid, and flavonoids) and a reduced dataset in which flavonoids were combined into enhancer and inhibitory sums to reduce collinearity; the reduced-dataset results are presented in the main text and the full-dataset results in the Supplementary data. A partial Spearman correlation controlling for Mo was used to test whether the Al–cellular iron uptake association was independent of co-varying inhibitors.

All statistical analyses and data visualizations were performed in MinutePlot® (version 1.0.0, Opimis AB, Gothenburg, Sweden; https://minuteplot.com).

## 3 Results and discussion

### 3.1 Extrudability of unrefined bean flours

Unrefined raw materials are inherently complex, comprising diverse cellular structures, macronutrients, micronutrients, and bioactive compounds that contribute to nutritional quality but also pose challenges for food processing (Germerdonk et al., 2025). Evaluating whether processing approaches such as low-moisture extrusion can reliably transform unrefined whole-pulse flour into nutrient-rich expanded snacks is therefore essential. In this context, extrudability serves as a key parameter for assessing how differences in raw material composition influence extrusion process performance and whether stable processing is achievable. In extrusion processing, extrudability is commonly quantified using specific mechanical energy (SME) and die pressure. SME reflects the mechanical energy transferred to the material per unit mass during extrusion, whereas die pressure describes the resistance of the molten material to flow through the die (Bouvier and Campanella, 2014; Guy, 2001). Together, these parameters are widely used indicators of extrusion process behavior and raw material extrudability (Luo et al., 2020; Sinaki et al., 2021).

Although identical extrusion conditions (e.g., barrel temperature profile, screw speed, and target moisture content) were applied across all flour types (Fig. 1a–f), significant differences in SME and die pressure were observed (Fig. 1g and h). These differences likely reflect variation in proximate composition (Table 1), as well as differences in protein and starch composition. High SME is generally associated with elevated melt viscosity (Singh et al., 2024). Navy, yellow, and red lentil flours exhibited the highest SME values, whereas chickpea flour showed the lowest (Fig. 1g). For yellow and navy bean flours, the elevated SME is consistent with their relatively high amylose contents reported in the literature (discussed further in Section 3.2), as high-amylose starches exhibit lower peak viscosity during pasting owing to restricted starch granule swelling compared with amylopectin-rich systems (Hooper et al., 2021; Hoover and Ratnayake, 2002; Tahir et al., 2011); this may relate to differences in starch swelling behavior, hydration kinetics, or barrel filling degree. Red lentil flour showed a comparably high SME despite its lower amylose content, indicating that amylose content alone does not fully explain the observed differences, and further work is required to disentangle these effects.

In contrast, chickpea flour exhibited lower SME despite its lower amylose content and higher pasting viscosity, which may be attributed to its comparatively high lipid content (6.0%). Lipids can act as lubricants within the extruder barrel, reducing friction and mechanical energy input (Hoover and Ratnayake, 2002; Stehle et al., 2024). Notably, differences in SME did not translate uniformly to die pressure across all flours, with red lentil and navy bean exhibiting the lowest die pressures. This decoupling may reflect variation in melt rheology along the barrel and differences in extensional viscosity at the die. Despite these compositional effects, the SME and die pressure values observed here fall within ranges previously reported for low-moisture extruded puffed snack products (Luo et al., 2020; Sinaki et al., 2021). Overall, these results demonstrate that unrefined bean flour can be successfully extruded into expanded snacks and that, under identical processing conditions, variation in flour composition leads to distinct extrudability responses.

### 3.2 Physical and textural properties of puffs

The extruded pulse puffs were evaluated for expansion ratio, hardness, and water activity, which are commonly used parameters for characterizing low-moisture extruded puffed snacks. Clear differences in all three properties were observed among puffs produced from different pulse cultivars (Fig. 2). Red lentil puffs exhibited the highest expansion ratio (6.44 ± 0.37), whereas chickpea puffs showed the lowest expansion (2.44 ± 0.15) (Fig. 2a). Yellow and navy bean puffs displayed moderate and comparable expansion ratios (4.31 ± 0.09 and 4.36 ± 0.06, respectively), while cowpea puffs exhibited intermediate but lower expansion (3.20 ± 0.01). As expected, expansion ratio was inversely related to hardness (Fig. 2b). Chickpea puffs, which expanded the least, exhibited the highest hardness (24.80 ± 1.03 N), whereas lentil puffs, with the greatest expansion, were the softest (1.60 ± 0.13 N). Yellow and navy bean puffs showed low hardness values (2.47 ± 0.14 N and 2.24 ± 0.05 N, respectively), indicating a favorable balance between expansion and mechanical strength. Water activity also differed significantly among samples (Fig. 2c) and was used as an indicator of crispiness, a key determinant of consumer acceptance in expanded snack products. Chickpea puffs exhibited the highest water activity (0.76), suggesting reduced crispiness and shorter potential shelf life, whereas navy, yellow, and lentil puffs exhibited lower values (0.52–0.57).

**Fig. 2:**
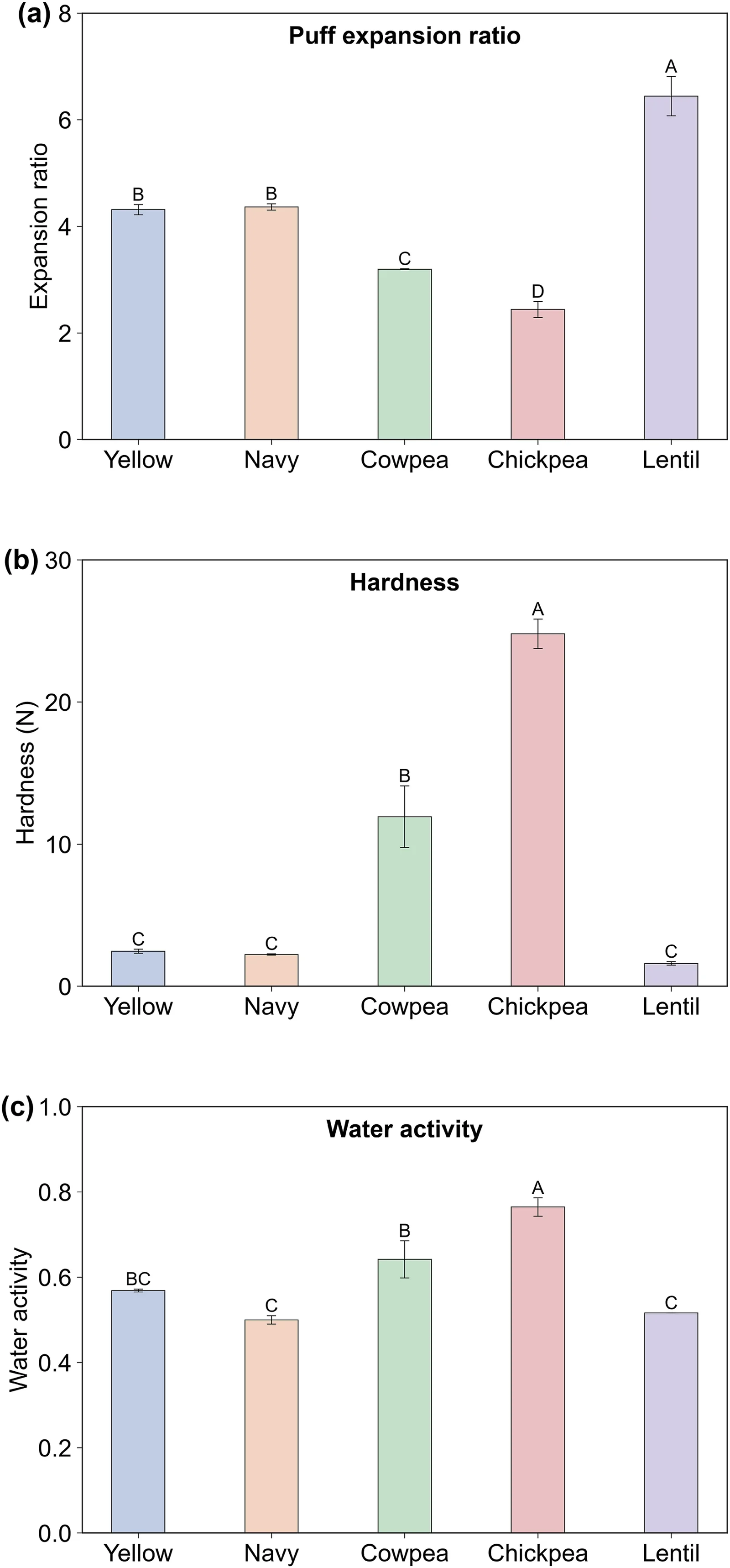
Comparison of expansion ratio (a), hardness (b), and water activity (c) of low-moisture extruded puffs produced from different pulse types. Results are reported on an as-is basis. Significant differences (*p* < 0.05) are denoted by different uppercase letters.

Although total starch contents were similar across all flours (Table 1), puff expansion may be influenced by starch molecular composition, particularly the amylose-to-amylopectin ratio. Higher amylopectin content generally promotes expansion, whereas elevated amylose content restricts expansion and yields denser structures (Vanier et al., 2016). Previous studies have shown that starches containing approximately 20–32% amylose exhibit greater expansion than high-amylose starches (Vanier et al., 2016). While amylose content was not measured in the present study, literature values indicate that the amylose contents of the pulse starches evaluated here fall within a comparable range (yellow bean: ∼31%; navy bean: 28–29%; cowpea: ∼26%; chickpea: 23–27%; red lentil: 23–25%) (Hoover and Ratnayake, 2002; Huang et al., 2007). These similarities suggest that differences in starch molecular composition are unlikely to be the primary drivers of the observed variation in puff expansion.

Instead, differences in puff structure and texture are more plausibly explained by variation in proximate composition, particularly lipid and fiber contents (Table 1). Chickpea flour contained substantially higher lipid levels (∼6%) than the other flours (<1%), and lipids are known to act as internal lubricants during extrusion, reducing SME and interfering with bubble formation (Jacquet et al., 2025). The combination of low SME and high lipid content likely contributed to the reduced expansion, increased hardness, and elevated water activity observed in chickpea puffs. In contrast, red lentil flour has a relatively low fiber content (8%) compared with navy bean (19%) and yellow bean (20%) (Table 1), which may have facilitated greater expansion, as elevated fiber levels are known to restrict product expansion (Ainsworth et al., 2007; Robin et al., 2012). Although the water activity values measured in this study exceeded those typically associated with optimal crispiness (<0.35) (Katz and Labuza, 1981), no post-extrusion drying was applied in order to isolate the effects of raw material composition. Overall, yellow and navy bean flour exhibited balanced expansion, low hardness, and relatively low water activity, performing comparably to red lentil, a pulse widely used in commercial puffed snack products. These results demonstrate the potential of unrefined yellow and navy bean flours as alternative raw materials for low-moisture extruded puffed snack production.

### 3.3 Phytic acid and lectin in pulse flours and their corresponding porridges and puffs

The capacity of foods to alleviate dietary iron deficiency depends in part on the presence of other compounds that could impair iron bioavailability, including phytate (phytic acid) and lectins; especially in beans and pulses that are not properly cooked (Petry et al., 2016; Piskin et al., 2022). Phytic acid, a major antinutrient in plant-based foods that chelates iron and reduces its bioavailability (Hart et al., 2015; Petry et al., 2010; Piskin et al., 2022), was therefore quantified in flours and their corresponding porridges and puffs to assess baseline levels and the effects of processing. Cowpea flour had the highest phytic acid concentrations when compared to the flour produced from other cultivar types (Fig. 3).

**Fig. 3:**
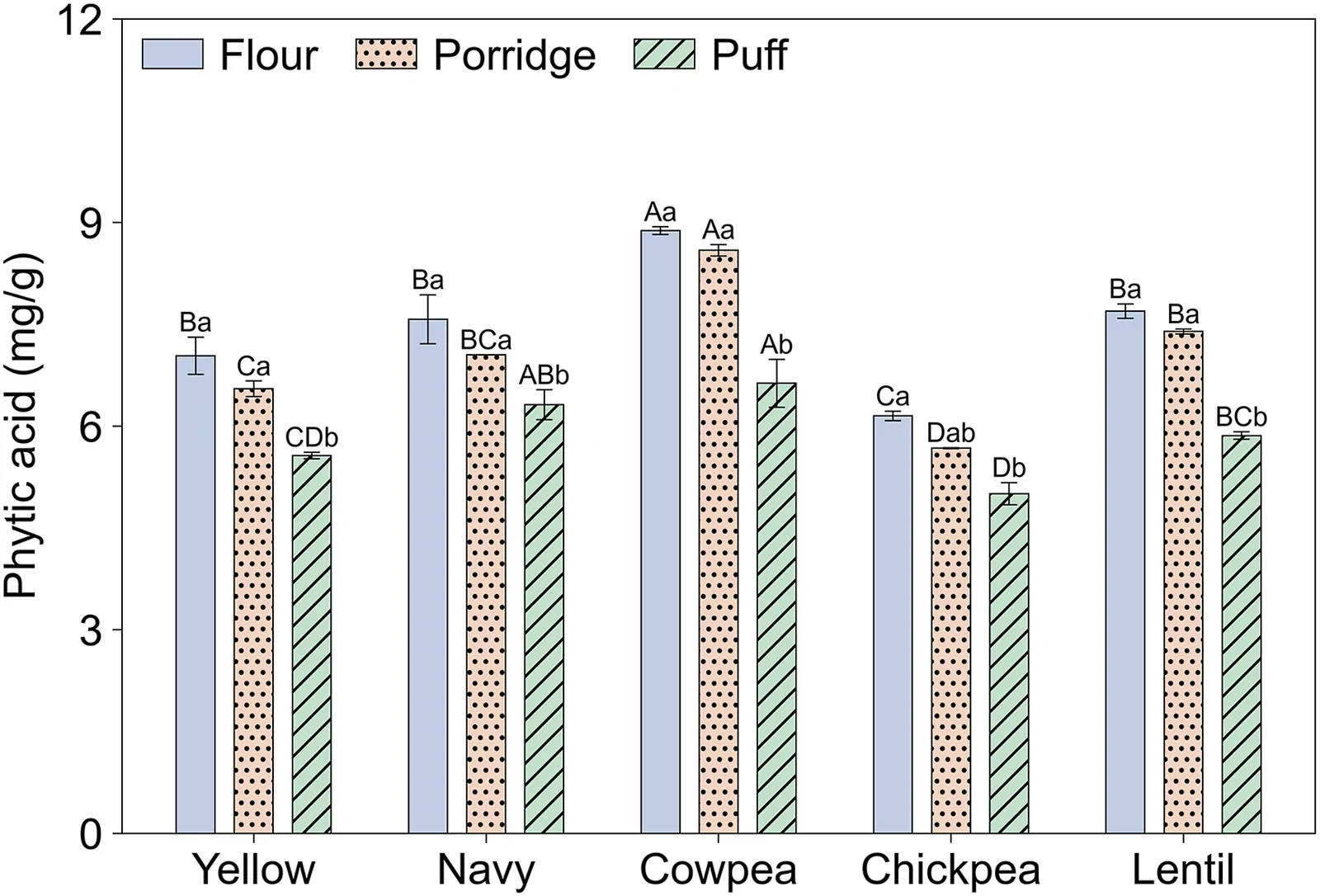
Phytic acid content of raw pulse flours and their corresponding porridges and expanded puffs. Results are reported on an as-is basis. Significant differences (*p* < 0.05) between processing methods (flour, porridge and puff) are denoted by different lowercase superscripts within each pulse type. Uppercase superscripts denote significant differences between pulse types processed into either 1) raw flour, 2) porridge, or 3) puff samples.

Puffs exhibited significantly lower phytic acid levels than their respective flours and porridges (Fig. 3). The greater reduction observed in puffs relative to porridges is consistent with the more intense conditions of low-moisture extrusion, which reduces phytic acid content in pulses (Alonso et al., 2000). However, phytate is relatively heat-stable, and the extent of its reduction depends on extrusion conditions, with both higher barrel temperature and higher feed moisture shown to significantly lower phytic acid rather than temperature alone (Abd El-Hady and Habiba, 2003). Nevertheless, the phytic acid concentrations in pulse porridges and puffs remain high, ranging from 5 to 9 mg/g. Cowpea flour, porridge and puffs had significantly higher phytic acid concentrations compared to the products formulated from yellow beans (Fig. 3). When the concentrations of phytic acid become too high, especially in cereals, there can be a substantial impairment on iron bioavailability (Hurrell, 2003; Schlemmer et al., 2009; Shi et al., 2018; Wiesinger et al., 2022).

Lectin activity was measured as an indicator of product safety, as residual active lectins can affect intestinal epithelial permeability and nutrient metabolism (Campion et al., 2009; Markell et al., 2017; Petry et al., 2016). Porridges were included as a reference treatment to enable comparison with extrusion, reflecting the fact that pulses are not consumed in their raw form, and porridge formulation undergoes an intense boiling treatment, which is used to inactivate lectins and other digestive inhibitors that are often found in unprocessed bean and pulse seeds (Wiesinger et al., 2022).

Hemagglutination activity, used as a measure of lectin activity, is commonly considered high at titers ≥1:256 and low at titers ≤1:2 (Adamcová et al., 2021; Nciri et al., 2015; Thompson et al., 2025). Accordingly, hemagglutination activity in the unrefined whole-pulse flours was generally high to moderately high, with the exception of the commercial chickpea flour, whereas hemagglutination activity was undetectable in all porridges (boiled) and in most extruded puffed products (**Supplementary data**; Table S1). These reductions are consistent with previous reports (Abd El-Hady and Habiba, 2003; Kelkar et al., 2012; Rehman and Shah, 2005) and reflect the greater heat sensitivity of lectins relative to phytic acid.

Residual phytic acid and lectin activity detected in cowpea puffs may reflect its initially higher phytic acid and lectin content combined with relatively low SME, which could limit complete inactivation and warrants further investigation. Overall, porridges and puffs produced from yellow and navy bean flours exhibited no detectable hemagglutination activity, indicating product safety, alongside substantially reduced phytic acid levels, with the greatest reductions observed in puffed products. Taken together, these results indicate that, when coupled with favorable iron and flavonoid profiles, yellow and navy bean-based extrudates have characteristics well-suited for use in expanded snack products.

### 3.4 Mineral concentrations

Although dietary promoters or inhibitors strongly influence iron bioavailability, adequate iron concentration remains a necessary prerequisite for effective iron delivery. Accordingly, the mineral concentrations of flours and their corresponding porridges and puffs were quantified.

Among the flours, yellow bean had the highest iron concentration and chickpea the lowest, with navy, cowpea, and lentil at intermediate levels; notably, chickpea flour, a common commercial puffed-snack ingredient, was significantly lower in iron than the bean and cowpea flours (Fig. 4a). For each flour type, iron concentrations did not differ significantly among flours, porridges, and puffs, indicating that processing did little to alter total iron. Despite similar iron levels, all puffed products exhibited significantly lower phytate-iron molar ratios than their corresponding flours and porridges, with yellow bean puffs showing the lowest ratios (Fig. 4b). Because phytate-to-iron molar ratios above 10 are associated with strong inhibition of iron absorption (Wiesinger et al., 2020; Glahn et al., 2002), the ratios observed in puffs (6–9) suggest reduced inhibition and thus a greater potential for improved iron bioavailability. The lower phytate-iron molar ratios in puffed products primarily reflect reductions in phytic acid achieved during high temperature, low-moisture extrusion (Fig. 3).

**Fig. 4:**
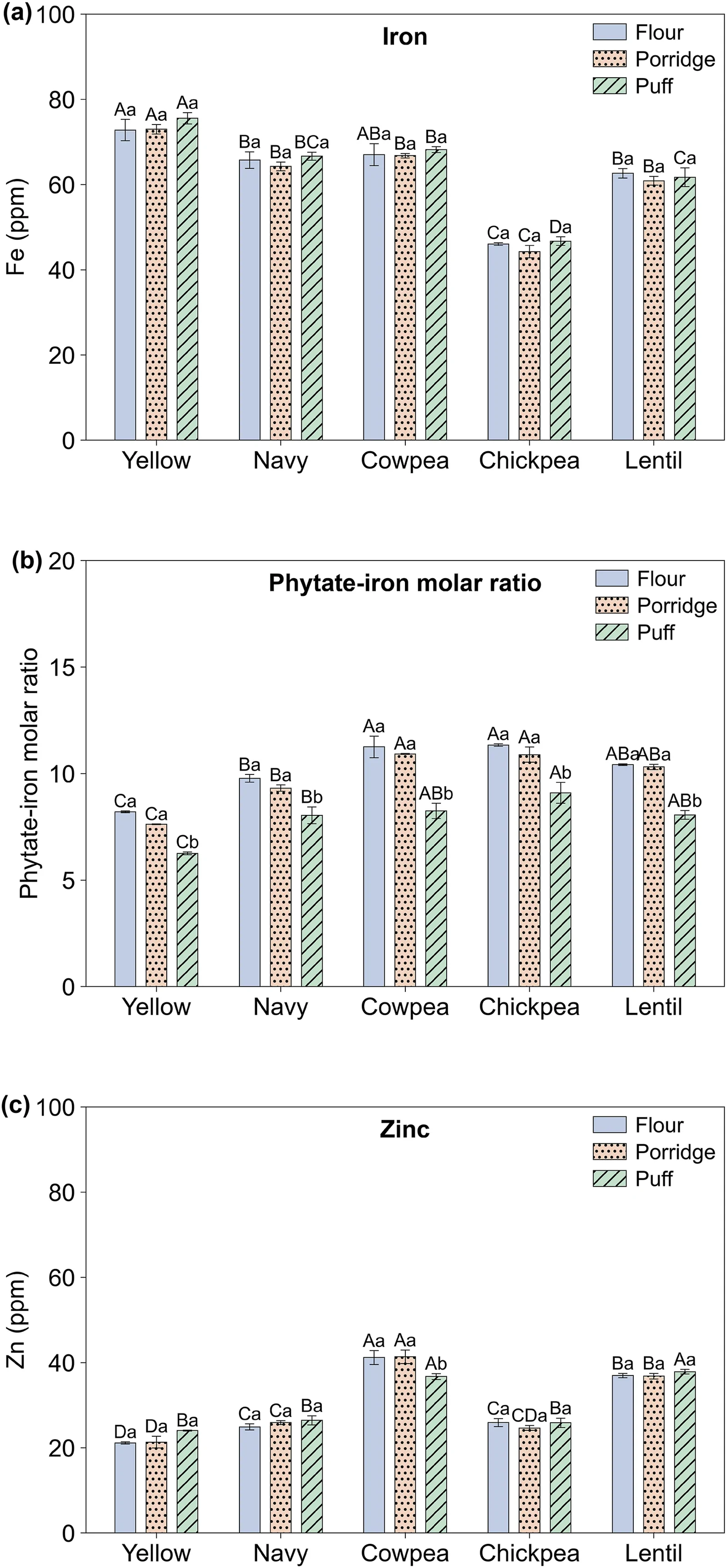
Iron (a), phytate-iron molar ratio (b), and zinc (c) content of raw pulse flours and their corresponding porridges and expanded puffs. Results are reported on an oven-dried weight basis. Significant differences (*p* < 0.05) between processing methods (flour, porridge and puff) are denoted by different lowercase superscripts within each pulse type. Uppercase superscripts denote significant differences between pulse types processed into either 1) raw flour, 2) porridge, or 3) puff samples.

Zinc, another essential dietary mineral, has been reported to compete with iron absorption when present at high, therapeutic doses (Kordas and Stoltzfus, 2004; Wiesinger et al., 2022). Yellow and navy bean flour, along with their corresponding porridges and puffs, contained significantly lower zinc concentrations than cowpea- and lentil-based products (Fig. 4c). Consistent with this, zinc was negatively associated with cellular iron uptake across the sample set (ρ = −0.72; Fig. 6), and the low zinc concentrations of yellow and navy bean products therefore align with their high iron uptake (discussed later). Furthermore, yellow and navy beans are rich in other nutritionally relevant minerals, including calcium, magnesium, manganese, and potassium (**Supplementary data**; Table S2). Processing had little effect on the concentrations of these minerals as well, further contributing to the extruded puffs’ overall nutritional value.

### 3.5 Flavonoids

Flavonoids are plant polyphenols concentrated primarily in the seed coats of beans and can be broadly classified as enhancers or inhibitors of iron bioavailability based on their effects on intestinal iron uptake (Hart et al., 2015, 2017, 2019). Several flavonoids, including kaempferol and its glycosides, epicatechin, and malvidin-3-glucoside, have been reported to enhance iron absorption, whereas quercetin and myricetin derivatives, gallocatechin, and several anthocyanins and proanthocyanidins act as inhibitors (Hart et al., 2015, 2017, 2019; Li et al., 2023). These contrasting effects arise from differences in iron-binding strength, redox behavior, and the stability of iron–flavonoid complexes under intestinal conditions (Lesjak et al., 2014, 2019; Li et al., 2023). Weakly chelating or reducing flavonoids may enhance iron bioavailability by maintaining iron solubility, whereas strongly chelating flavonoids, particularly those bearing a pyrogallol (3′,4′,5′-trihydroxy) B-ring (e.g., myricetin, delphinidin, and gallocatechin), form stable, non-transportable complexes that inhibit absorption (Hart et al., 2015, 2017, 2019; Lesjak et al., 2014, 2019; Wiesinger et al., 2020). Glycosylation further modulates this behavior because the principal iron-binding site of flavonols lies at the 3-hydroxyl group: 3-O-glycosylation (as in kaempferol 3-glucoside and kaempferol 3-sambubioside) tends to weaken chelation and preserve iron solubility, thereby limiting the inhibition of iron uptake (Lesjak et al., 2014), whereas flavonoids with a pyrogallol B-ring chelate iron strongly and form stable, poorly absorbed complexes that reduce its bioavailability (Li et al., 2023). Notably, even small amounts of inhibitory flavonoids can outweigh the benefits of enhancers; for example, inclusion of only 10% myricetin in a myricetin:epicatechin (10:90) mixture reduced iron bioavailability by ∼65% compared with epicatechin alone (Hart et al., 2017). These structure-dependent differences provide a mechanistic basis for interpreting the flavonoid profiles quantified here in relation to cellular iron uptake. Accordingly, both enhancer and inhibitory flavonoids were quantified in this study (Table 4).

**Table 4:** Flavonoid contents (nmol/g; freeze-dried weight basis) of raw pulse flours and their corresponding porridges and expanded puffs. All 25 targeted flavonoids were analyzed across all samples; none of the 25 flavonoids selected for analysis were detected in chickpea flour or in products derived from it. *nd*: not detected. Significant differences (*p* < 0.05) are denoted by different lowercase superscripts within each pulse type.

Flavonoids	Yellow			Navy			Cowpea			Lentil		
	Flour	Porridge	Puff	Flour	Porridge	Puff	Flour	Porridge	Puff	Flour	Porridge	Puff
Enhancers of iron uptake												
Kaempferol	56.33 ± 2.52^a^	33.72 ± 5.65^b^	39.96 ± 0.49^b^	*nd*	*nd*	*nd*	*nd*	*nd*	*nd*	*nd*	*nd*	*nd*
Kaempferol 3-glucoside	777 ± 37.49^a^	693 ± 36.44^a^	492 ± 33.74^b^	5.47 ± 0.01^a^	3.12 ± 0.49^b^	1.04 ± 0.01^c^	*nd*	*nd*	*nd*	*nd*	*nd*	*nd*
Kaempferol 3-sambuioside	264 ± 17.15^a^	154 ± 16.09^b^	127 ± 0.76^b^	3.77 ± 0.23^a^	3.45 ± 0.18^a^	2.08 ± 0.19^b^	*nd*	*nd*	*nd*	*nd*	*nd*	*nd*
Catechin	*nd*	*nd*	*nd*	*nd*	*nd*	*nd*	8.13 ± 0.43^a^	6.38 ± 0.47^a^	6.80 ± 0.67^a^	*nd*	*nd*	*nd*
Epicatechin	*nd*	*nd*	*nd*	*nd*	*nd*	*nd*	1.86 ± 0.94^a^	0.88 ± 0.10^a^	0.64 ± 0.07^a^	*nd*	*nd*	*nd*
Malvidin 3-glucoside	*nd*	*nd*	*nd*	*nd*	*nd*	*nd*	12.82 ± 0.58^a^	10.40 ± 0.04^b^	2.29 ± 0.38^c^	*nd*	*nd*	*nd*

Inhibitors of iron uptake												
Quercetin	*nd*	*nd*	*nd*	*nd*	*nd*	*nd*	2.41 ± 0.21^a^	0.88 ± 0.11^b^	1.08 ± 0.07^b^	*nd*	*nd*	*nd*
Quercetin 3-glucoside	1.03 ± 0.04	*nd*	*nd*	*nd*	*nd*	*nd*	14.89 ± 0.20^a^	8.51 ± 0.72^b^	5.34 ± 0.34^c^	*nd*	*nd*	*nd*
Quercetin 3-rutinoside	0.83 ± 0.33	*nd*	*nd*	0.99 ± 0.01^a^	0.85 ± 0.03^b^	0.61 ± 0.02^c^	0.91 ± 0.08^a^	0.74 ± 0.06^a^	0.66 ± 0.04^a^	*nd*	*nd*	*nd*
Myricetin	*nd*	*nd*	*nd*	*nd*	*nd*	*nd*	*nd*	*nd*	*nd*	*nd*	*nd*	*nd*
Myricetin 3-glucoside	*nd*	*nd*	*nd*	*nd*	*nd*	*nd*	19.14 ± 1.13^a^	9.26 ± 0.47^b^	5.98 ± 0.16^c^	1.58 ± 0.63^a^	0.92 ± 0.10^a^	0.67 ± 0.15^a^
Gallocatechin	*nd*	*nd*	*nd*	*nd*	*nd*	*nd*	0.97 ± 0.03^a^	0.84 ± 0.08^a^	0.77 ± 0.04^a^	*nd*	*nd*	*nd*
Cyanidin 3-glucoside	*nd*	*nd*	*nd*	*nd*	*nd*	*nd*	4.40 ± 0.41^a^	2.93 ± 0.73^ab^	1.01 ± 0.08^b^	*nd*	*nd*	*nd*
Delphinidin 3-glucoside	*nd*	*nd*	*nd*	*nd*	*nd*	*nd*	6.20 ± 0.26^a^	4.77 ± 0.59^ab^	3.56 ± 0.39^b^	*nd*	*nd*	*nd*
Petunidin 3-glucoside	*nd*	*nd*	*nd*	*nd*	*nd*	*nd*	8.22 ± 0.16^a^	6.00 ± 0.33^b^	3.57 ± 0.31^c^	*nd*	*nd*	*nd*

Yellow bean flour and its corresponding porridges and puffs were enriched in enhancer flavonoids, particularly kaempferol and its glycosides, whereas navy bean products contained substantially lower levels (Table 4). Although processing reduced flavonoid concentrations across all samples, likely due to thermal degradation (Crozier et al., 1997), puffs produced from yellow bean flour retained higher levels of kaempferol and its glycosides than navy bean puffs. Cowpea flour contained a different enhancer profile, with moderate levels of catechin, epicatechin, and malvidin 3-glucoside rather than kaempferol-type compounds, whereas neither chickpea nor red lentil contained any of the analyzed enhancer flavonoids (Table 4). Especially lentil flour is typically made from dehulled seeds, which may explain the absence.

In contrast, inhibitory flavonoids were present only at trace levels in yellow and navy bean flours, limited to quercetin derivatives (quercetin 3-glucoside in yellow; quercetin 3-rutinoside in both yellow and navy), and were markedly reduced after processing into porridges and puffs (Table 4). Cowpea flour, however, contained high levels of inhibitory flavonoids with a pyrogallol B-ring, including myricetin 3-glucoside, delphinidin 3-glucoside, and petunidin 3-glucoside. Although reduced in porridges and puffs, these inhibitory compounds in addition to quercetin 3-glucoside remained higher than in all other samples. Chickpea contained none of the analyzed inhibitory flavonoids, while red lentil contained only trace amounts of myricetin-3-glucoside. Overall, the combination of minimal inhibitory flavonoids and abundant enhancer flavonoids in yellow and navy bean puffs underscores their potential to enhance iron bioavailability, as discussed in the following section.

### 3.6 Cellular iron uptake

Cellular iron uptake, assessed by Caco-2 cell ferritin formation, was significantly higher in porridges and puffs produced from yellow bean flour than in those from all other pulse types (Fig. 5). For yellow bean, processing into porridge and puffs significantly increased cellular iron uptake relative to the flour, likely reflecting disruption of cotyledon cell walls and improved access of digestive enzymes to iron (Wiesinger et al., 2020), consistent with previous reports (Hooper et al., 2023). Similar processing-related improvements were observed for navy bean and chickpea (Fig. 5). Although the effect of low-moisture extrusion on cellular iron uptake in yellow and navy bean products was not statistically significant relative to porridge processing, higher processing temperatures are generally associated with enhanced potential iron bioavailability (Hooper et al., 2023). This effect was evident for chickpea and red lentil, where puffs exhibited significantly higher cellular iron uptake than their corresponding porridges, indicating that low-moisture extrusion can enhance potential iron bioavailability relative to porridge processing.

**Fig. 5:**
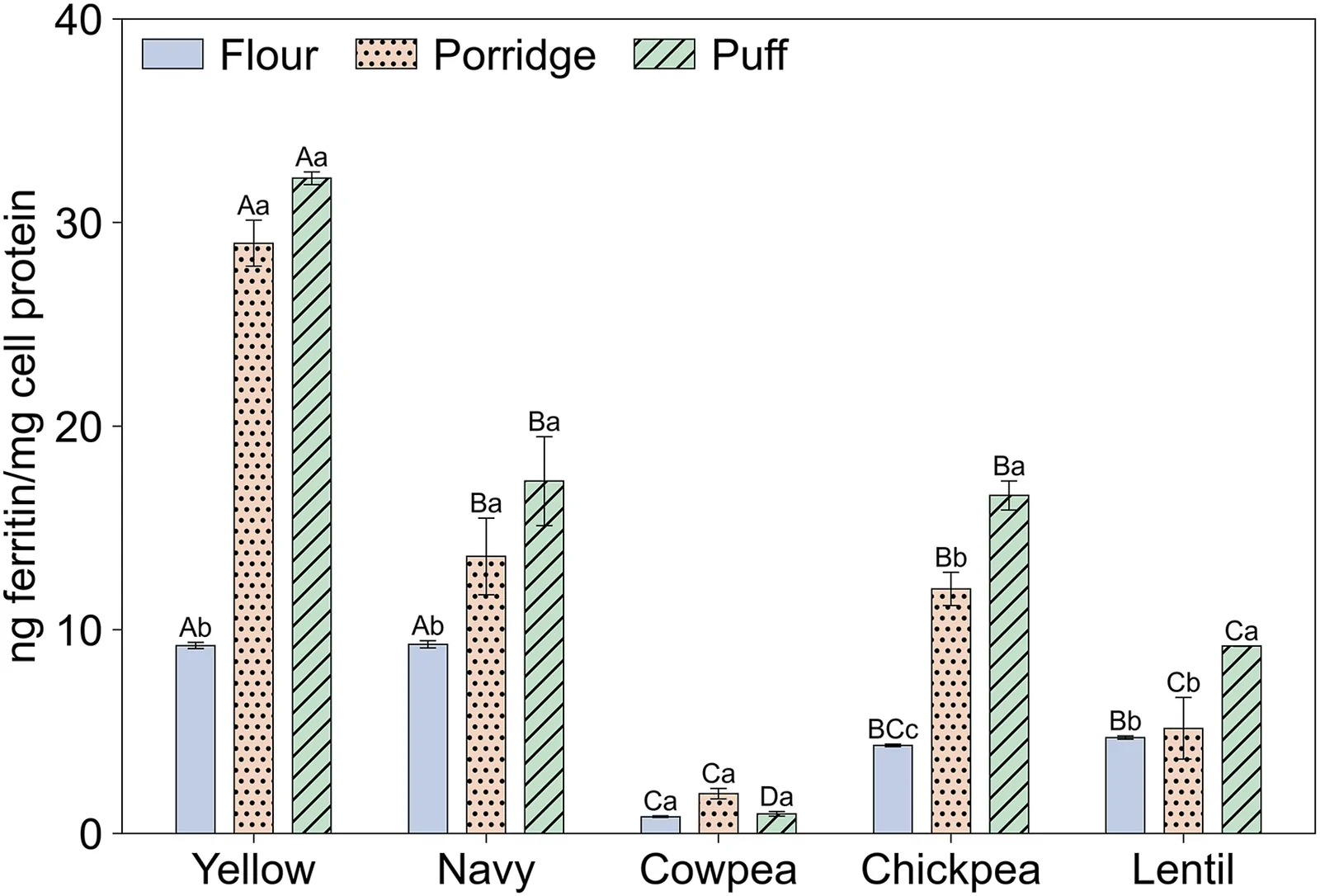
Cellular iron uptake, expressed as Caco-2 cell ferritin formation (ng ferritin/mg cell protein), in raw pulse flours and their corresponding porridges and expanded puffs. Results are reported on a freeze-dried weight basis. Individual replicate values are provided in the Supplementary data. Significant differences (*p* < 0.05) between processing methods (flour, porridge and puff) are denoted by different lowercase superscripts within each pulse type. Uppercase superscripts denote significant differences between pulse types processed into either 1) raw flour, 2) porridge, or 3) puff samples.

Across the different pulse types, yellow bean products exhibited the highest cellular iron uptake, followed by navy bean products. Notably, iron concentration alone did not predict cellular iron uptake: navy and cowpea puffs contained similar iron levels, yet navy puffs exhibited significantly higher cellular iron uptake, and chickpea puffs outperformed cowpea puffs despite their lower iron content (Fig. 4; Fig. 5). These observations indicate that the food matrix and its composition after processing, rather than total iron, govern cellular iron uptake; the specific compositional factors underlying these differences are examined in the following section.

When compared with previously reported products, the cellular iron uptake of navy and chickpea puffs was comparable to that of low-moisture extruded pasta produced from white kidney bean flour (Hooper et al., 2023). In this study, yellow bean porridges and puffs had higher cellular iron uptake compared to low-moisture extruded white kidney bean pasta run in the same Caco-2 bioassay (Fig. 5; Table 3). An *in vivo* study demonstrated that white kidney bean pasta, as part of a meal with tomato sauce, vegetables and cabbage, is a good source of bioavailable iron, especially compared to black bean pasta with high concentrations of inhibitory flavonoids, including delphinidin 3-glucoside and petunidin 3-glucoside. The white kidney bean pasta was produced with flour that had broken cotyledon cell walls and no detectable iron-inhibiting flavonoids, such as quercetin, myricetin, delphinidin and procyanidin B1 (Glahn et al., 2025). Unlike the porridges and puffs formulated from the yellow beans, white kidney bean pasta only has nominal amounts of iron-bioavailability-promoting kaempferol compounds (Glahn et al., 2025).

Cooked ground beef and Impossible™ meat were also included to give perspective on how the cellular iron uptake of the expanded puff products compares to foods derived from other animal- and plant-based ingredients. Ferritin values for yellow bean porridge and puffs (∼30 ng/mg total cell protein) were significantly lower than the ferritin values in Caco-2 cells exposed to an *in vitro* digestion of ground beef and Impossible™ meat (44–49 ng/mg total cell protein) (Fig. 5, Table 3). These reference foods are considered highly bioavailable sources of iron. Although the ferritin response to yellow bean porridges and puffs was significantly lower than that of beef and the Impossible™ product, it nonetheless represents a comparatively high response within this assay for iron derived from a single plant ingredient. This comparison reflects the cellular ferritin response under the conditions of the *in vitro* digestion/Caco-2 model and should not be interpreted as indicating equivalent iron absorption in humans, given the substantial differences in iron form, concentration, and matrix among these foods. Addition of other iron absorption enhancers, including vitamin C, could further increase the cellular iron uptake of the yellow bean porridges and puffs beyond what was observed in this study. Moreover, iron-promoting ingredients that improve the taste, texture, and storage capacity of the expanded puffs could be explored in a future study, again creating ready-to-eat products (snacks, cereals, and pasta) with enhanced potential iron bioavailability.

Iron bioavailability values generated using the Caco-2 cell model are inherently matrix dependent and influenced by factors such as iron dose and cell status; therefore, absolute values are most appropriately interpreted relative to comparable food matrices rather than as universal thresholds (Glahn, 2009; Glahn et al., 1998, 2016). Viewed in this context, the consistently higher ferritin formation observed for yellow bean products reinforces the central role of food matrix composition in governing potential iron bioavailability beyond iron concentration alone. Overall, these findings identify yellow bean and its derived products as particularly promising for enhancing potential iron bioavailability. The yellow bean market class evaluated in this study was Manteca, with a pale-yellow seed coat color. This market class was previously formulated into a dried, shelf-stable pasta product with significantly higher iron concentrations and iron bioavailability than those of enriched and whole wheat pasta, as well as other commercially available chickpea, lentil, and gluten-free pastas made from maize and rice (Wiesinger et al., 2023). It is important to note that substantial variation exists among yellow bean market classes, and not all market classes are expected to confer similarly high iron bioavailability (Sadohara et al., 2025; Wiesinger et al., 2018). While porridges can support iron intake, their preparation is less standardized and often context dependent; in contrast, puffed snacks produced from yellow bean flour provide a consistent, ready-to-eat format for improving dietary iron delivery.

### 3.7 Correlation and multivariate analysis

Spearman correlation (Fig. 6) and principal component analysis (Fig. 7) were used to identify the compositional factors most closely associated with cellular iron uptake, and both analyses converge on the same conclusion: cellular iron uptake was governed chiefly by the level of iron-absorption inhibitors rather than by iron content. Cellular iron uptake correlated negatively with phytic acid (ρ = −0.62), Zn (ρ = −0.72), and the summed inhibitory flavonoids (ρ = −0.75) (Fig. 6), which reflects the capacity of strongly chelating flavonoids with a pyrogallol B-ring to form stable, poorly absorbed iron complexes (Hart et al., 2015; Lesjak et al., 2014; Li et al., 2023), and is consistent with the roles of phytate and zinc as recognized inhibitors of non-heme iron absorption (Hurrell, 2003; Kordas and Stoltzfus, 2004; Wiesinger et al., 2020). Phosphorus closely followed phytic acid, while Mo (ρ = −0.62) and S (ρ = −0.52) also correlated negatively with cellular iron uptake; however, these associations most likely reflect the co-variation of Mo and S with the principal inhibitors in the inhibitor-rich cowpea samples rather than independent effects. Aluminum also showed a positive correlation with uptake (ρ = 0.64), but this is interpreted as a spurious, confounded association: it co-varied inversely with Mo and S and became non-significant after adjustment for Mo (partial ρ = 0.45; **Supplementary data**, Fig. S2). Furthermore, aluminum has been reported to compete with iron for transferrin binding and to impair iron bioavailability (Donnelly et al., 1990; Trapp, 1983), making a direct enhancing effect biologically implausible. By contrast, total iron was not significantly correlated with cellular iron uptake (ρ = 0.12), reinforcing that iron concentration alone does not determine bioavailability (Piskin et al., 2022). Consistent with these patterns, the PCA biplot separated the inhibitor-rich cowpea samples from the yellow and navy bean samples (Fig. 7), with the latter having the lowest inhibitor flavonoid levels and the highest cellular iron uptake.

**Fig. 6:**
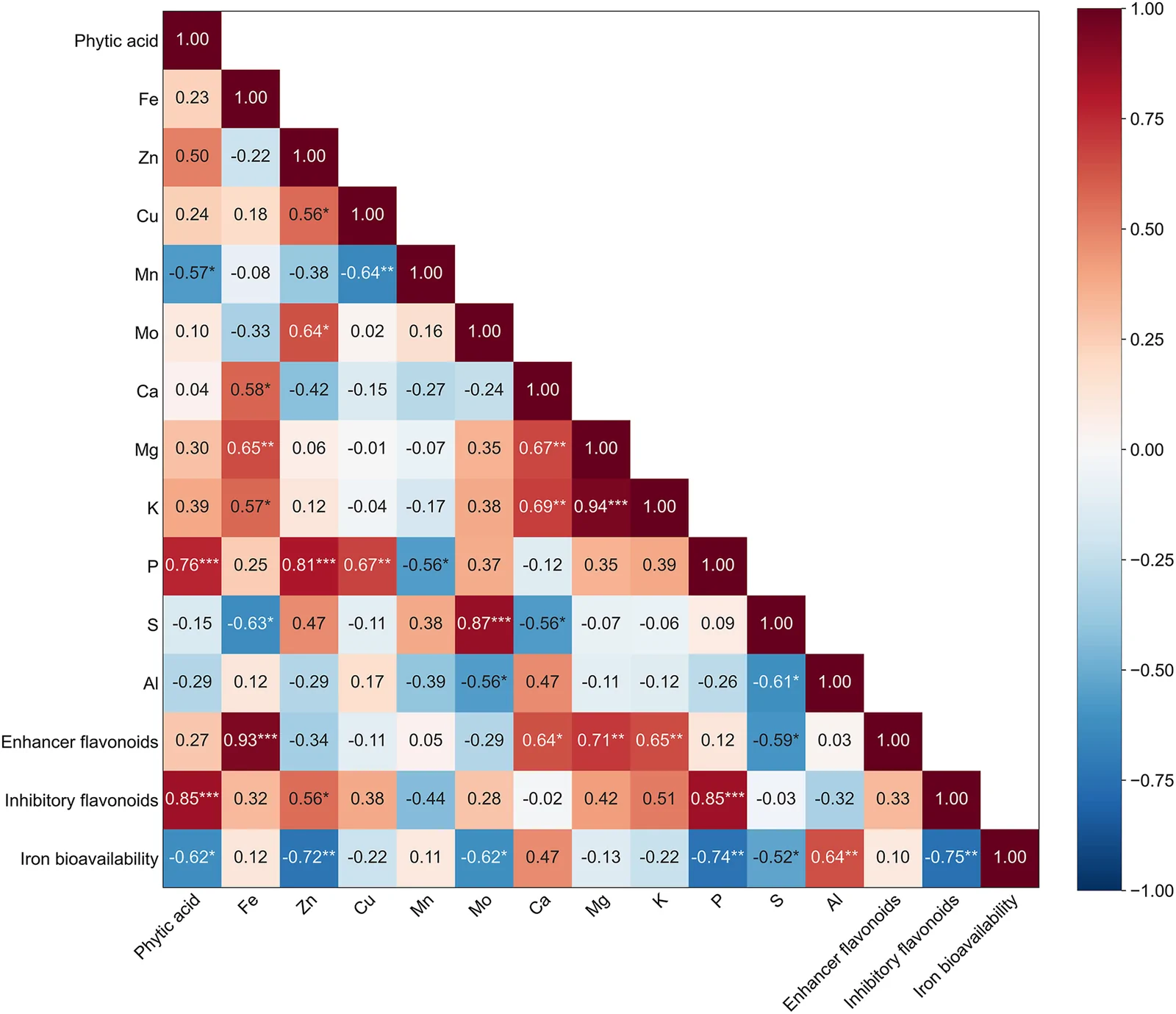
Spearman correlation matrix of mineral, phytate, and flavonoid composition and iron bioavailability across five pulse types (Yellow, Navy, Cowpea, Chickpea, Lentil), each processed into flour, porridge, and puff. Enhancer and inhibitory flavonoids were each expressed as the sum of their individual compounds to reduce collinearity and sparsity. Cell values are correlation coefficients; color intensity reflects the strength and direction of correlation (red, positive; blue, negative). Asterisks denote significance (*p < 0.05, **p < 0.01, ***p < 0.001).

**Fig. 7:**
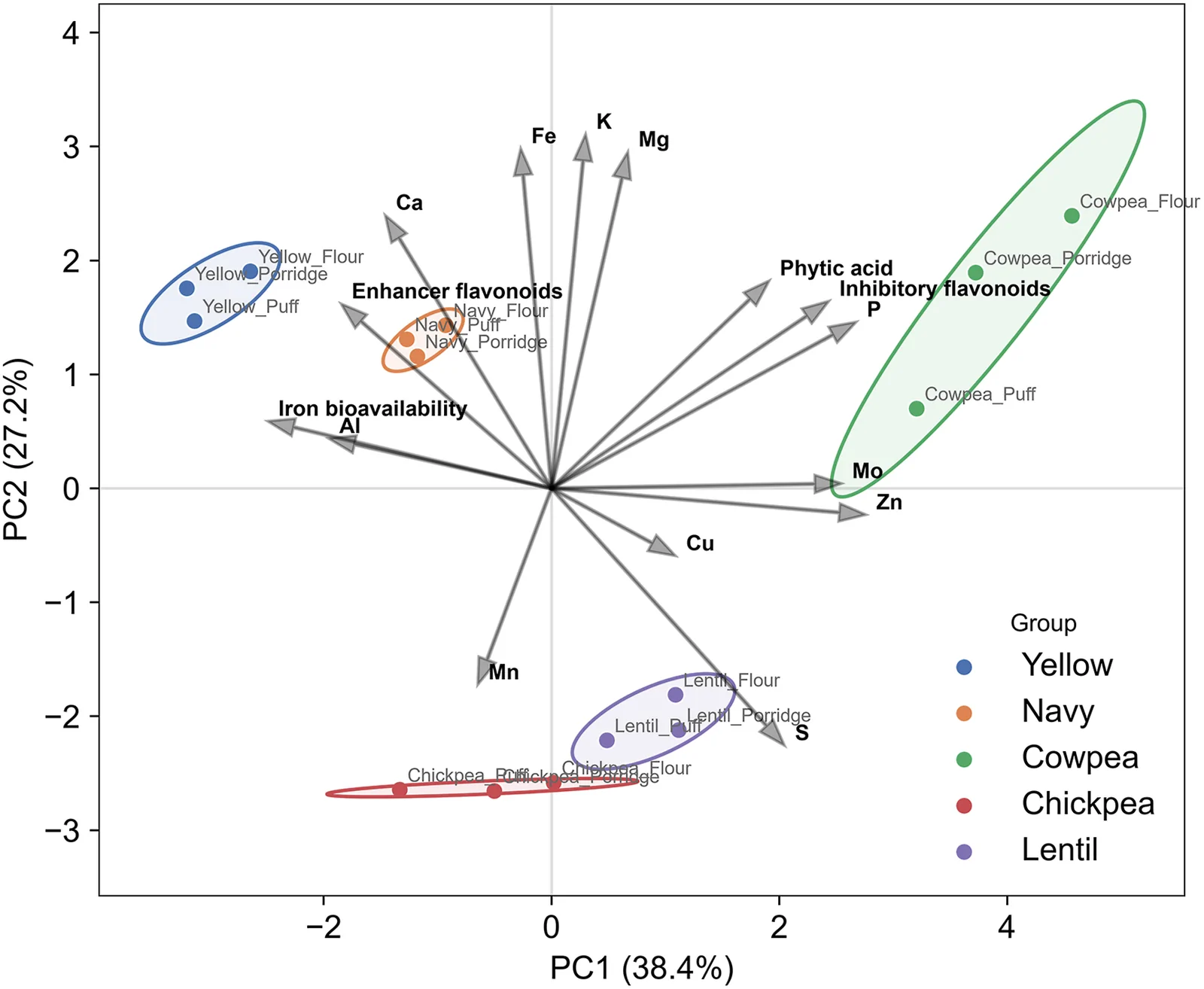
Principal component analysis (PCA) biplot relating mineral, phytate, and flavonoid composition to iron bioavailability across five pulse types (Yellow, Navy, Cowpea, Chickpea, Lentil), each processed into flour, porridge, and puff. Enhancer and inhibitory flavonoids were each expressed as the sum of their individual compounds to reduce collinearity and sparsity. Points are samples colored by pulse type and grouped with 95% confidence ellipses; arrows are variables, where direction indicates correlation with the principal components (PC1 and PC2) and length reflects contribution. Variables pointing the same way are positively correlated, and opposite ways negatively correlated. PC1 and PC2 explained 38.4% and 27.2% of the variance, respectively.

The summed enhancer flavonoids were likewise uncorrelated with cellular iron uptake (ρ = 0.10), indicating that enhancer flavonoid content did not translate into a measurable benefit in these samples. The individual enhancer flavonoids could not be assessed in isolation, as each was detected almost exclusively within a single pulse type and was consequently highly intercorrelated (ρ up to ≈1.00 among the catechin and anthocyanin compounds of cowpea; **Supplementary data**, Fig. S1), and no association remained once they were combined into class sums (Fig. 6). Taken together, the high cellular iron uptake of yellow bean products is best explained by a favorable combination of compositional factors: a near-absence of inhibitory flavonoids, abundant enhancer flavonoids, and a comparatively low phytate-to-iron molar ratio (Wiesinger et al., 2020). Enhancer flavonoids such as kaempferol and its glycosides and epicatechin can promote non-heme iron uptake by keeping iron soluble and absorbable (Hart et al., 2015, 2017; Li et al., 2023), so their abundance in yellow bean is favorable. This benefit is conditional, however, because even small amounts of strongly chelating inhibitory flavonoids can outweigh it, as shown by the ∼65% reduction in iron uptake when only 10% myricetin was added to epicatechin (Hart et al., 2017). Therefore, the net effect of a flavonoid profile thus depends on the balance between enhancer and inhibitory flavonoids rather than on enhancer abundance alone (Hart et al., 2017; Li et al., 2023). A low inhibitor level alone, however, was not sufficient for high uptake: red lentil contained few inhibitory flavonoids yet showed only modest cellular iron uptake, consistent with its lower enhancer flavonoid content and less favorable phytate-to-iron ratio. High cellular iron uptake therefore appears to require a low level of inhibitory flavonoids acting together with abundant enhancers and a favorable phytate-to-iron ratio, as realized in yellow bean, rather than any single factor alone.

### 3.8 Implications for improving plant-based iron delivery at scale

The high cellular iron uptake observed for yellow bean puffs highlights the potential of matrix-optimized, ready-to-eat pulse-based foods to contribute meaningfully to improving iron status at scale. A theoretical intake of approximately 93–357 g of yellow bean puffs, based on their iron content, would be sufficient to meet daily iron requirements (7–27 mg) across population groups, assuming that the high cellular iron uptake measured *in vitro* translates into comparable absorption in humans, which remains to be confirmed. In typical dietary contexts, a daily intake of ∼30–50 g, corresponding to a standard snack serving, could provide a nutritionally relevant contribution to iron intake, especially if the iron is highly bioavailable. This intake assumes that iron is also obtained from other dietary sources and that the presence of enhancer flavonoids from yellow bean puffs may further support iron uptake from co-consumed foods.

Beyond standalone snacks, yellow bean flour offers flexibility for incorporation into commonly consumed food formats (Fig. 8). In the context of plant-based meat alternatives, inclusion of ∼35% yellow bean flour, leaving ∼65% formulation space for other functional ingredients, would allow meaningful integration without compromising product design. At this inclusion level, yellow bean flour could provide amounts of iron comparable to lean meat cuts on a weight-for-weight basis (Pretorius et al., 2016; Valenzuela et al., 2009). Further, yellow bean flour contains ∼17% protein and ∼20% dietary fiber (Table 1), which will also help increase the overall nutritive value of the food product. However, as with all composite foods, the overall food matrix, including interactions with other ingredients, will ultimately determine iron bioavailability and warrants further investigation.

**Fig. 8:**
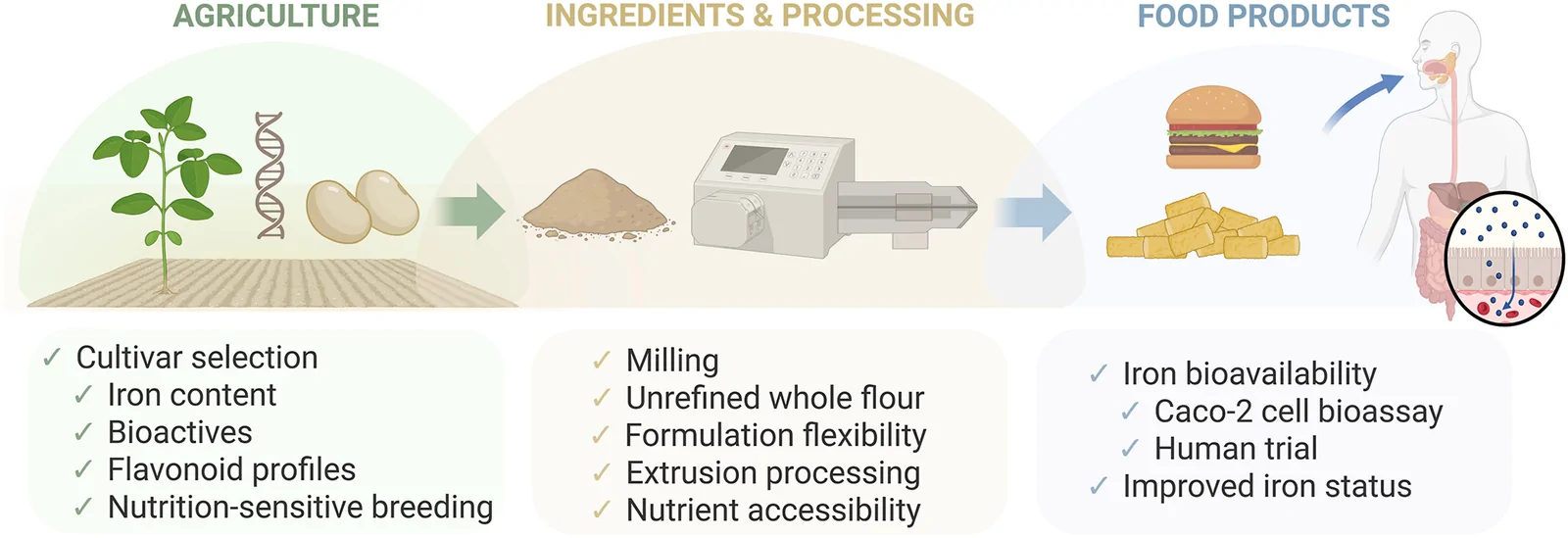
Conceptual schematic illustrating how nutrition-sensitive crop selection, use of unrefined ingredients, processing strategies that accommodate raw-material complexity, and incorporation into culturally relevant foods can jointly support improved iron delivery and iron status at the population level. Figure created with BioRender.com.

Several limitations of this study should be acknowledged. Iron bioavailability was assessed using the *in vitro* digestion/Caco-2 cell ferritin model, a widely used and validated indicator of iron bioavailability (Glahn, 2009; Glahn et al., 1998, 2016); however, this model measures cellular iron uptake rather than transepithelial iron transport, and does not directly estimate human fractional iron absorption, which is further influenced by whole-meal composition and dietary inhibitors such as phytates and oxalates. The phenolic analysis was targeted rather than untargeted, so unquantified matrix components may also have contributed to the observed responses. In addition, starch fine structure (amylose-to-amylopectin ratio) was not measured directly but inferred from published values for these pulses; direct determination would help confirm its contribution to the observed differences in extrudability and puff expansion. Further, extrusion was performed in a limited number of independent trials per flour type, and the associations reported here, while internally consistent across correlation and multivariate analyses, are based on a small sample size. Future work should therefore confirm these findings in animal and human intervention studies, extend the compositional profiling to untargeted phenolic and other matrix constituents, and evaluate yellow bean ingredients within complete meals and composite formulations.

Dietary guidance that emphasizes minimizing strong iron absorption inhibitors at iron-rich meals, together with clearer labeling of bioavailable iron content, could further support effective iron delivery to consumers. In addition, screening of locally adapted pulse cultivars and nutrition-sensitive breeding for favorable iron–polyphenol profiles offer scalable pathways to extend these benefits to regions where yellow beans are not readily grown (Fig. 8). Together, these findings support scalable strategies to improve plant-based iron delivery across populations, while promoting the use of unrefined whole-pulse flours and contributing to more sustainable food systems (Germerdonk et al., 2025).

## 4 Conclusion

This study evaluated the nutritional composition and cellular iron uptake of unrefined pulse flours produced in the United States (yellow bean, navy bean, cowpea, chickpea and lentil) and assessed the feasibility of producing convenient, ready-to-eat puffed snacks from these flours using low-moisture extrusion to address dietary iron deficiency. Flour composition strongly influenced extrudability, with higher lipid content reducing SME and puff expansion; chickpea flour produced dense, hard puffs, whereas yellow and navy bean flours extruded similarly and yielded puffs with comparable texture. Across pulses, cellular iron uptake was most closely associated with the balance of iron-absorption inhibitors and promoting factors rather than with iron content alone: correlation and multivariate analyses indicated that high iron uptake required a low level of inhibitory flavonoids together with a favorable iron context, namely a low phytate-to-iron molar ratio and the presence of enhancer flavonoids. A low inhibitor level alone was not sufficient; red lentil, for example, contained few inhibitory flavonoids yet showed only modest uptake, consistent with its lower enhancer content and less favorable phytate-to-iron ratio. Yellow bean puffs showed the highest cellular iron uptake and cowpea puffs showed the lowest, with extrusion providing a modest improvement relative to porridge processing. Low-moisture extrusion is well suited to this application, offering a scalable, standardized process that converts compositionally complex, unrefined pulse flours into shelf-stable, ready-to-eat snacks. Puff properties such as expansion and crispiness could be further improved through formulation strategies, including incorporating amylopectin-rich starches and applying post-extrusion drying, and through optimization of extrusion conditions, while breeding for reduced dietary fiber may further enhance expansion and cellular iron uptake. Incorporating iron-absorption enhancers, such as kaempferol-rich ingredients and vitamin C, may provide an additional route to increase potential iron bioavailability. Together, these findings highlight matrix-sensitive ingredient selection, combined with processing that tolerates raw-material complexity, as a critical lever for improving iron bioavailability in plant-based foods and advancing scalable, sustainable plant-based iron delivery.

## Data availability

Data will be made available on request.

## Declaration of competing interest

The authors declare the following financial interests/personal relationships which may be considered as potential competing interests: MS reports a relationship with Opimis AB that includes: equity or stocks. MinutePlot®, used for data processing and statistical analysis in this study, is developed and distributed by Opimis AB, of which MS is a founder. The other authors declare no competing interests. If there are other authors, they declare that they have no known competing financial interests or personal relationships that could have appeared to influence the work reported in this paper.

## Figures and Tables

**Fig. S1:**
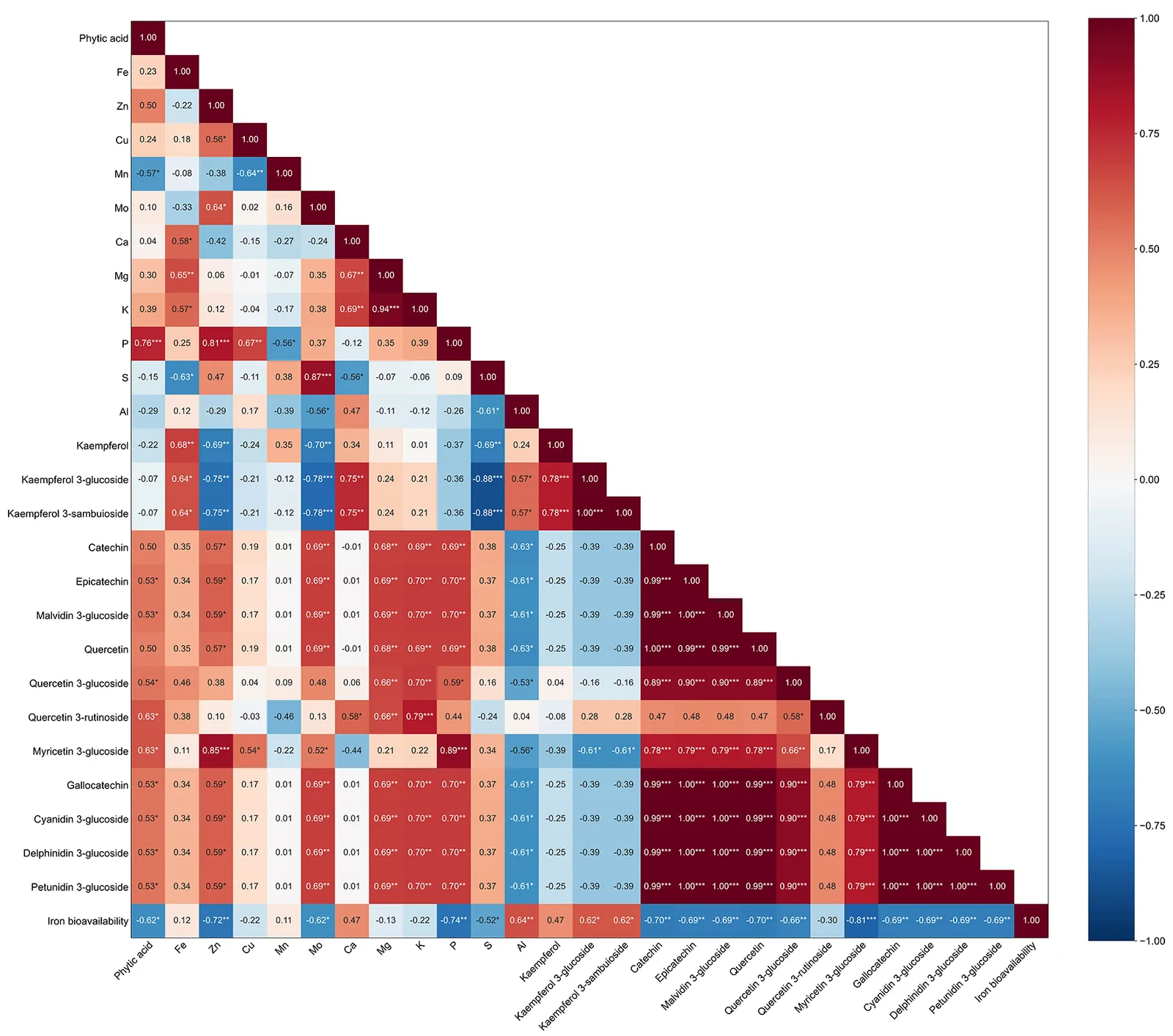
Spearman correlation matrix of mineral, phytate, and individual flavonoid composition and iron bioavailability across five pulse types (Yellow, Navy, Cowpea, Chickpea, Lentil), each processed into flour, porridge, and puff. All individual flavonoids were included as separate variables; the catechin and anthocyanin compounds were strongly intercorrelated (ρ ≈ 0.99–1.00) as they were detected in a limited number of samples, and were combined into enhancer and inhibitory sums in the main-text analysis (Fig. X). Cell values are correlation coefficients; color intensity reflects the strength and direction of correlation (red, positive; blue, negative). Asterisks denote significance (*p < 0.05, **p < 0.01, ***p < 0.001).

**Fig. S2:**
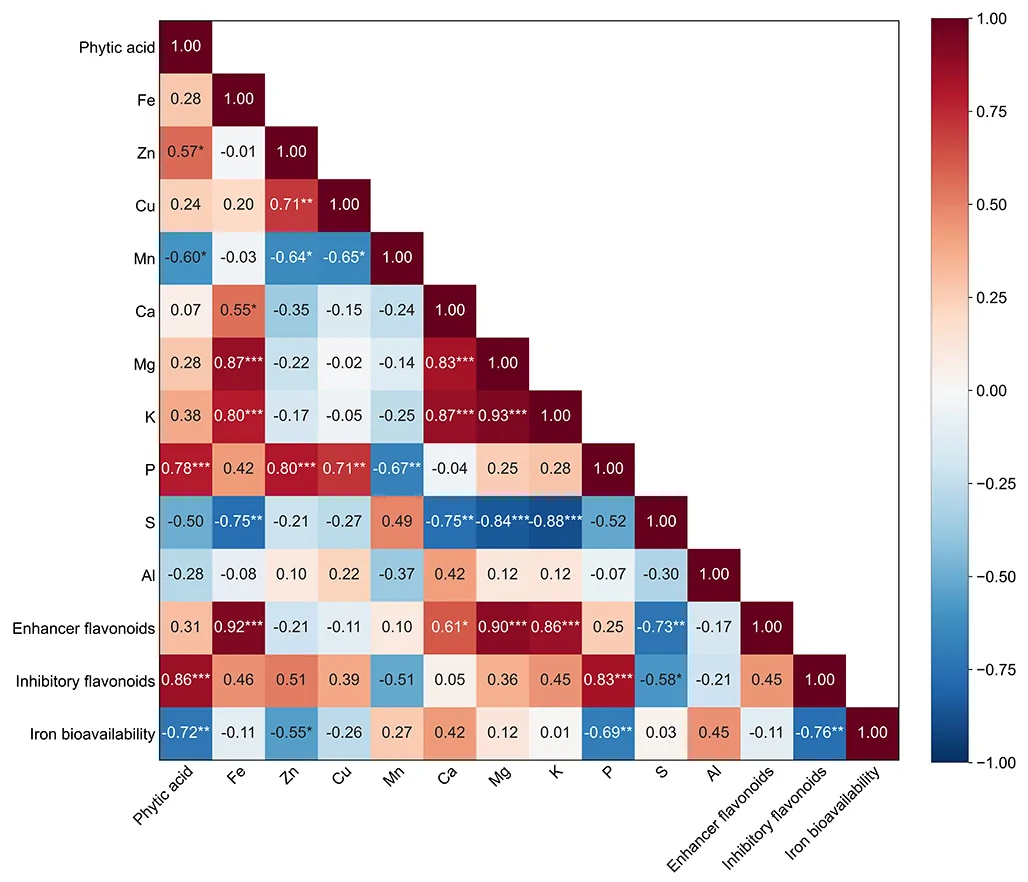
Partial Spearman correlation matrix of mineral, phytate, and flavonoid composition and iron bioavailability across five pulse types (Yellow, Navy, Cowpea, Chickpea, Lentil), each processed into flour, porridge, and puff, controlling for molybdenum (Mo). Enhancer and inhibitory flavonoids were each expressed as the sum of their individual compounds. Cell values are partial correlation coefficients; color intensity reflects the strength and direction of correlation (red, positive; blue, negative). Asterisks denote significance (*p < 0.05, **p < 0.01, ***p < 0.001). Adjustment for Mo reduced the positive aluminum (Al)–iron bioavailability association from ρ = 0.64 in the unadjusted matrix (Fig. 6) to a non-significant partial ρ = 0.45.

**Fig. S3:**
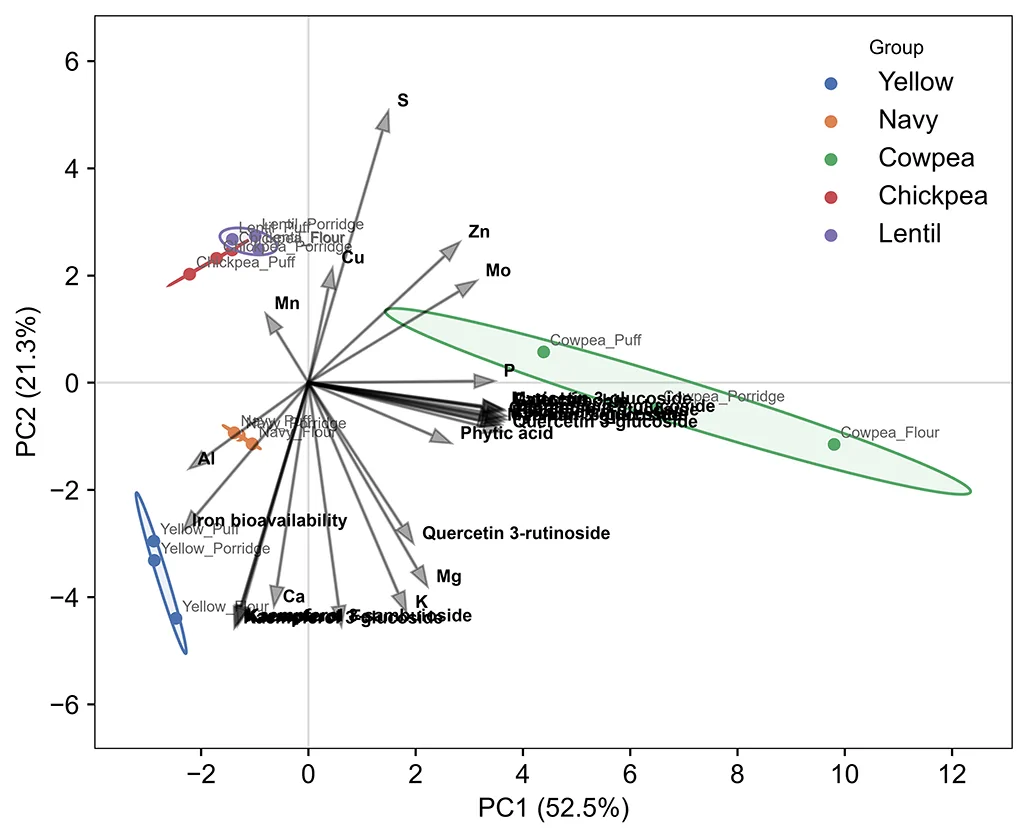
Principal component analysis (PCA) biplot relating mineral, phytate, and flavonoid composition to iron bioavailability across five pulse types (Yellow, Navy, Cowpea, Chickpea, Lentil), each processed into flour, porridge, and puff. All individual flavonoids were included as separate variables. Points are samples colored by pulse type and grouped with 95% confidence ellipses; arrows are variables, where direction indicates correlation with the principal components (PC1 and PC2) and length reflects contribution. Variables pointing the same way are positively correlated, and opposite ways negatively correlated. PC1 and PC2 explained 52.5% and 21.3% of the variance, respectively.
