# Supplementary material for: Matrix-controlled iron bioavailability in unrefined whole-pulse low-moisture extrudates
Source: Curr Res Food Sci. 2026 Sep 30;13:101589. doi: 10.1016/j.crfs.2026.101589 (PMC13677430; doi:10.1016/j.crfs.2026.101589)
Supplement: Supplementary file 1 [file mmc1.docx]

**Supporting data**

**Table S1**. lectin activity, expressed as hemagglutination activity, of raw bean, chickpea, and lentil flours and their corresponding porridges and expanded puffs.

|  | Yellow bean | | |  | Navy bean | | |  | Cowpea bean | | |  | Chickpea | | |  | | Lentil | |
| --- | --- | --- | --- | --- | --- | --- | --- | --- | --- | --- | --- | --- | --- | --- | --- | --- | --- | --- | --- |
|  | **Flour** | **Porridge** | **Puff** |  | **Flour** | **Porridge** | **Puff** |  | **Flour** | **Porridge** | **Puff** |  | **Flour** | **Porridge** | **Puff** |  | **Flour** | **Porridge** | **Puff** |
| Hemagglutination Activity | 1:512 | *nd* | *nd* |  | 1:256 | *nd* | *nd* |  | 1:16 | *nd* | 1:2 |  | *nd* | *nd* | *nd* |  | 1:128 | *nd* | *nd* |

Hemagglutination activity was expressed as the reciprocal of the highest dilution showing visible agglutination, with higher titers indicating greater lectin activity; *nd* refers to no agglutination (i.e., no detectable lectin activity).

**Table S2**. Mineral contents (ppm) of raw bean flours and their corresponding porridges and puffs.

|  | **Yellow** | | | **Navy** | | | **Cowpea** | | | **Chickpea** | | | **Red lentil** | | |
| --- | --- | --- | --- | --- | --- | --- | --- | --- | --- | --- | --- | --- | --- | --- | --- |
|  | Flour | Porridge | Puff | Flour | Porridge | Puff | Flour | Porridge | Puff | Flour | Porridge | Puff | Flour | Porridge | Puff |
| Cu | 6.2 | 6.5 | 7.2 | 6.7 | 7.1 | 8.1 | 6.5 | 7.5 | 10.6 | 5.6 | 6.1 | 6.2 | 8.8 | 9 | 11.5 |
| Mn | 16.8 | 16.5 | 17.7 | 10.7 | 10.5 | 11.3 | 14.5 | 14.2 | 14.3 | 25.1 | 25.4 | 25.1 | 12.1 | 11.8 | 12.3 |
| Mo | 0.5 | 0.5 | 0.9 | 1.6 | 1.6 | 2.6 | 5.8 | 6 | 5.9 | 3.6 | 3.6 | 3.4 | 1.6 | 1.6 | 2.4 |
| Ca | 1607.6 | 1616.3 | 1629.2 | 2164.3 | 2200.4 | 2203.5 | 1105 | 1143 | 1092.2 | 1019.7 | 1058 | 1053.7 | 423.4 | 423.1 | 445.1 |
| Mg | 1653 | 1725.3 | 1729.3 | 1792.8 | 1608.3 | 1829 | 2140.5 | 2215.5 | 1954.3 | 1365.2 | 1414 | 1376.5 | 920.5 | 933.6 | 954.6 |
| K | 11447.8 | 11311 | 11421.1 | 11502.2 | 11520.8 | 11901.2 | 12509.8 | 12339.9 | 11936.9 | 10154.2 | 10166.9 | 9787.5 | 8464.1 | 8376.2 | 9025 |
| P | 3384.5 | 3287 | 3480.9 | 3660.9 | 3579.9 | 3600.1 | 5005.4 | 4617.6 | 4567.5 | 3306.6 | 3157.3 | 3057.5 | 3950.2 | 3856.3 | 3839.5 |
| S | 1104.5 | 1121 | 1202.4 | 1392 | 1325.9 | 1406.1 | 1564.5 | 1582.4 | 1599.8 | 1716.4 | 1677.3 | 1590.5 | 1487.3 | 1550.6 | 1561.6 |
| Al | 9 | 8 | 9.4 | 10 | 9.1 | 11.3 | 5.7 | 6.9 | 5.6 | 6.9 | 6.6 | 8.7 | 7.4 | 6.9 | 10.4 |

**Table S3**. Two-way ANOVA summary for phytic acid in pulse extrudates, testing the effects of pulse type (Yellow, Navy, Cowpea, Chickpea, Lentil), processing type (flour, porridge, puff), and their interaction. Sum of squares (SS), degrees of freedom (df), F-values, and p-values are reported for each term (α = 0.05; n = 2 processing replicates per treatment).

| **term** | **SS** | **df** | **F** | **p** |
| --- | --- | --- | --- | --- |
| Pulse type | 19.061 | 4 | 158.138 | < 0.0001 |
| Processing | 13.64 | 2 | 226.328 | < 0.0001 |
| Pulse type:Processing | 1.398 | 8 | 5.799 | 0.0018 |
| Residual | 0.452 | 15 |  |  |

**Table S4**. Effect sizes (η², partial η², and ω²) with corresponding magnitude classifications for the two-way ANOVA of phytic acid in pulse extrudates, for the effects of pulse type, processing type, and their interaction.

| **term** | **η²** | **partial η²** | **ω²** | **magnitude** |
| --- | --- | --- | --- | --- |
| Pulse type | 0.552 | 0.977 | 0.548 | large |
| Pulse type × Processing | 0.04 | 0.756 | 0.033 | small |
| Processing | 0.395 | 0.968 | 0.393 | large |

**Table S5**. Two-way ANOVA summary for iron in pulse extrudates, testing the effects of pulse type (Yellow, Navy, Cowpea, Chickpea, Lentil), processing type (flour, porridge, puff), and their interaction. Sum of squares (SS), degrees of freedom (df), F-values, and p-values are reported for each term (α = 0.05; n = 2 processing replicates per treatment).

| **term** | **SS** | **df** | **F** | **p** |
| --- | --- | --- | --- | --- |
| Pulse type | 2658.716 | 4 | 305.107 | < 0.0001 |
| Processing | 18.795 | 2 | 4.314 | 0.0331 |
| Pulse type:Processing | 8.515 | 8 | 0.489 | 0.8459 |
| Residual | 32.678 | 15 |  |  |

**Table S6**. Effect sizes (η², partial η², and ω²) with corresponding magnitude classifications for the two-way ANOVA of iron in pulse extrudates, for the effects of pulse type, processing type, and their interaction.

| **term** | **η²** | **partial η²** | **ω²** | **magnitude** |
| --- | --- | --- | --- | --- |
| Pulse type | 0.978 | 0.988 | 0.974 | large |
| Pulse type × Processing | 0.003 | 0.207 | ~0 | negligible |
| Processing | 0.007 | 0.365 | 0.005 | negligible |

**Table S7**. Two-way ANOVA summary for the phytate-to-iron molar ratio in pulse extrudates, testing the effects of pulse type (Yellow, Navy, Cowpea, Chickpea, Lentil), processing type (flour, porridge, puff), and their interaction. Sum of squares (SS), degrees of freedom (df), F-values, and p-values are reported for each term (α = 0.05; n = 2 processing replicates per treatment).

| **term** | **SS** | **df** | **F** | **p** |
| --- | --- | --- | --- | --- |
| Pulse type | 35.584 | 4 | 129.285 | < 0.0001 |
| Processing | 29.211 | 2 | 212.256 | < 0.0001 |
| Pulse type:Processing | 1.658 | 8 | 3.012 | 0.0314 |
| Residual | 1.032 | 15 |  |  |

**Table S8**. Effect sizes (η², partial η², and ω²) with corresponding magnitude classifications for the two-way ANOVA of the phytate-to-iron molar ratio in pulse extrudates, for the effects of pulse type, processing type, and their interaction.

| **term** | **η²** | **partial η²** | **ω²** | **magnitude** |
| --- | --- | --- | --- | --- |
| Pulse type | 0.527 | 0.972 | 0.523 | large |
| Pulse type × Processing | 0.025 | 0.616 | 0.016 | small |
| Processing | 0.433 | 0.966 | 0.43 | large |

**Table S9**. Two-way ANOVA summary for zinc in pulse extrudates, testing the effects of pulse type (Yellow, Navy, Cowpea, Chickpea, Lentil), processing type (flour, porridge, puff), and their interaction. Sum of squares (SS), degrees of freedom (df), F-values, and p-values are reported for each term (α = 0.05; n = 2 processing replicates per treatment).

| **term** | **SS** | **df** | **F** | **p** |
| --- | --- | --- | --- | --- |
| Pulse type | 1480.456 | 4 | 429.297 | < 0.0001 |
| Processing | 0.193 | 2 | 0.112 | 0.895 |
| Pulse type:Processing | 43.687 | 8 | 6.334 | 0.0011 |
| Residual | 12.932 | 15 |  |  |

**Table S10**. Effect sizes (η², partial η², and ω²) with corresponding magnitude classifications for the two-way ANOVA of zinc in pulse extrudates, for the effects of pulse type, processing type, and their interaction.

| **term** | **η²** | **partial η²** | **ω²** | **magnitude** |
| --- | --- | --- | --- | --- |
| Pulse type | 0.963 | 0.991 | 0.96 | large |
| Pulse type × Processing | 0.028 | 0.772 | 0.024 | small |
| Processing | 0 | 0.015 | ~0 | negligible |

**Table S11**. Two-way ANOVA summary for cellular iron uptake in pulse extrudates, testing the effects of pulse type (Yellow, Navy, Cowpea, Chickpea, Lentil), processing type (flour, porridge, puff), and their interaction. Sum of squares (SS), degrees of freedom (df), F-values, and p-values are reported for each term (α = 0.05; n = 2 processing replicates per treatment).

| **term** | **SS** | **df** | **F** | **p** |
| --- | --- | --- | --- | --- |
| Pulse type | 1666.721 | 4 | 469.004 | < 0.0001 |
| Processing | 482.094 | 2 | 271.316 | < 0.0001 |
| Pulse type:Processing | 380.502 | 8 | 53.535 | < 0.0001 |
| Residual | 13.327 | 15 |  |  |

**Table S12**. Effect sizes (η², partial η², and ω²) with corresponding magnitude classifications for the two-way ANOVA of cellular iron uptake in pulse extrudates, for the effects of pulse type, processing type, and their interaction.

| **term** | **η²** | **partial η²** | **ω²** | **magnitude** |
| --- | --- | --- | --- | --- |
| Pulse type | 0.656 | 0.992 | 0.654 | large |
| Pulse type × Processing | 0.15 | 0.966 | 0.147 | large |
| Processing | 0.19 | 0.973 | 0.189 | large |

**Table S13**. Individual replicate values for cellular iron uptake, expressed as Caco-2 cell ferritin formation (ng ferritin/mg cell protein), in raw pulse flours and their corresponding porridges and expanded puffs. (r1 and r2 refer to two independent replicates)

|  | **Flour** | | **Porridge** | | **Puff** | |
| --- | --- | --- | --- | --- | --- | --- |
|  | **r1** | **r2** | **r1** | **r2** | **r1** | **r2** |
| **Yellow** | 9.11 | 9.33 | 28.18 | 29.77 | 32.39 | 31.95 |
| **Navy** | 9.41 | 9.15 | 14.93 | 12.26 | 18.84 | 15.75 |
| **Cowpea** | 0.84 | 0.79 | 2.12 | 1.76 | 0.88 | 1.03 |
| **Chickpea** | 4.36 | 4.27 | 11.41 | 12.57 | 17.09 | 16.08 |
| **Lentil** | 4.76 | 4.64 | 4.08 | 6.22 | 9.20 | 9.18 |
